# Comparative Efficacy and Safety of 11 Drugs as Therapies for Adults With Neuropathic Pain After Spinal Cord Injury: A Bayesian Network Analysis Based on 20 Randomized Controlled Trials

**DOI:** 10.3389/fneur.2022.818522

**Published:** 2022-03-21

**Authors:** Hai-Qian Ling, Zi-Hao Chen, Lei He, Feng Feng, Chuang-Gui Weng, Si-Jin Cheng, Li-Min Rong, Pei-Gen Xie

**Affiliations:** ^1^Department of Spine Surgery, The Third Affiliated Hospital of Sun Yat-sen University, Guangzhou, China; ^2^Guangdong Provincial Center for Quality Control of Minimally Invasive Spine Surgery, Guangzhou, China; ^3^Guangdong Provincial Center for Engineering and Technology Research of Minimally Invasive Spine Surgery, Guangzhou, China; ^4^Department of Orthopedics, Longgang Orthopedics Hospital of Shenzhen, Shenzhen, China

**Keywords:** neuropathic pain, spinal cord injury, network meta-analysis, efficacy, safety

## Abstract

**Objective:**

To provide an updated analysis of the efficacy and safety of drugs for the management of neuropathic pain (NP) after spinal cord injury (SCI) based on Bayesian network analysis.

**Methods:**

A Bayesian network meta-analysis of literature searches within PubMed, Cochrane Library, Embase, and Web of Science databases from their inception to February 21 2021 was conducted without language restrictions. Paired and network meta-analyses of random effects were used to estimate the total standardized mean deviations (SMDs) and odds ratios (ORs).

**Results:**

A total of 1,133 citations were identified and 20 RCTs (including 1,198 patients) involving 11 drugs and placebos for post-SCI NP selected. The 5 outcomes from all 11 drugs and placebos had no inconsistencies after Bayesian network analysis. BTX-A gave the most effective pain relief for the 4 weeks, following a primary outcome. No significant differences were found among drugs with regard to adverse events of the primary outcome. Gabapentin, BTX-A, and pregabalin were found to be the most helpful in relieving secondary outcomes of mental or sleep-related symptoms with differences in SMDs, ranging from −0.63 to −0.86. Tramadol triggered more serious adverse events than any of the other drugs with differences in ORs ranging from 0.09 to 0.11.

**Conclusion:**

BTX-A, gabapentin, pregabalin, amitriptyline, ketamine, lamotrigine, and duloxetine were all effective for NP management following SCI. Lamotrigine and gabapentin caused fewer side effects and had better efficacy in relieving mental or sleep-related symptoms caused by SCI-related NP. Tramadol, levetiracetam, carbamazepine, and cannabinoids could not be recommended due to inferior safety or efficacy.

**Systematic Review Registration:**

[https://inplasy.com/inplasy-2020-7-0061/], identifier [INPLASY202070061].

## Introduction

Spinal cord injury (SCI) is a rare event with devastating consequences and an estimated average global incidence of 23 cases per million (1) and a prevalence of between 236 and 1,298 per million (2). Patients present with a range of functional impairments, including sensory, motor, and autonomic dysfunctions, depending on the location and severity of the injury (3). Debilitating chronic neuropathic pain (NP) tends to affect 40% of patients following SCI (4–6) and represents a highly disabling clinical condition (7). As a result, treatment of post-SCI NP is vital to mitigate the impact on body function and overall quality of life.

Anticonvulsants, antidepressants, analgesics, and cannabinoids have all been used to treat NP (8–11), but refractory pain following SCI is common (12). Despite the modest short-term benefits of drug therapy, the balance between long-term benefits and damage has been overlooked. Clinical decision-makers and pharmaceutical enterprises have interests in the suitability of drugs for pain relief, making a comparison of drug efficacy and safety valuable. Most studies to date have been limited by manpower and material resources and have thus compared individual drugs with placebo or, at most, compared two drugs. Little valid data can be derived from direct comparisons, stimulating a longstanding debate about drug efficacy and safety (13).

Network meta-analysis extends pairwise meta-analyses to enable the pooling of data from many clinical trials, comparing at least two treatments. Inferences regarding the relative efficacy of each treatment are thus reinforced by the inclusion of both direct and indirect information (14, 15). A network meta-analysis based on Bayes' theorem of existing datasets provides a framework for comprehensive evaluation of drug efficacy and safety (16). Twenty RCTs were included in the present study, and Bayesian network analysis was performed to compare the efficacy and safety of different drugs for the treatment of NP in adults following SCI. The objective was to perform an evidence-based analysis for the benefit of clinical practitioners.

## Methods

### Data Sources and Study Strategy

A literature search of PubMed, Cochrane Library, Embase, and Web of Science from inception to February 21, 2021 was conducted without language restrictions. The subject words “spinal cord injury,” “neuropathic pain,” “treatment,” and “randomized controlled trials” were used. Subject words were combined with related free words (from Mesh or Emtree). The search strategies used in PubMed and Embase are shown in Supplementary Data 1, 2.

Study inclusion criteria were as follows: (1) single or double-blinded [non-blinded assessors of subjective measurement scale outcomes in RCTs generate substantially biased effect sizes (17)] randomized controlled trials (RCTs) that compared drugs with a placebo or a second drug; (2) patients were adults (≥ 18 years old) with SCI (rated from A to D according to the American Spinal Injury Association (ASIA) impairment scale; (3) patients had been diagnosed with NP according to the criteria of the International Association for the Study of Pain guidelines: (a) the onset of pain within 1 year following SCI; (b) no primary relation of pain to movement, inflammation or other local tissue damage; (c) use of adjectives ‘hot-burning,” “tingling,” “pricking,” “pins and needles,” “sharp,” “shooting,” “squeezing,” “cold,” “electric” or “shock like” to describe the quality of pain; (d) persistent pain for at least 1 month; (e) assessment of pain intensity using numeric rating scale (NRS ≥ 4) or visual analog scale (VAS ≥ 4; 0–10 cm) or VAS ≥ 40; 0–100 mm) where 0 = no pain and 10 or 100 = unbearable pain, respectively. Exclusion criteria were as follows: studies including non-drug therapy and non-RCTs; abstracts; studies with incomplete data, redundancy, or insufficient raw data; case reports; reviews; and meta-analyses.

Two researchers conducted independent screenings of titles and abstracts of the following retrieval. Thereafter, both researchers read the full texts, extracted data, and engaged in the discussion to arrive at a joint decision on validity for inclusion. A formal file extraction form was developed. The following data were extracted: author, subjects, sample size, average age, sex, intervention and control measures, follow-up time, intervention evaluation tools, and study design.

### Selection Criteria and Study Design

Primary outcomes were efficacy (a standardized pain score at 4 weeks) and safety (adverse events). Secondary outcomes included efficacy (a standardized pain score at 8 weeks), standardized mental or sleep-related assessment scores, and incidence of serious adverse events as supplemental safety outcomes (Supplementary Tables 1–5). Pain, mental, and sleep-related assessment differences were defined as the score of intensity changes (rating scales shown in Table 1) from the baseline to the end point. Patients' assessments for overall treatment were included among the secondary outcomes with lower scores on the specified rating scale, signifying better overall assessments. Some studies used more than one rating scale, necessitating standardization of continuous data. Results were recorded as close as possible to the 4-week follow-up time point for all analyses. If data were unavailable at the 4-week time point, data from between 3 and 17 weeks were used (time points close to 4 weeks were prioritized for primary outcomes and over 8 weeks for secondary outcomes).

**Table 1:** Baseline characteristics of included RCTs.

**Ref. no**.	**Author & year of publication**	**Country**	**Drugs** **intervention (I) & comparison (C)**	**Of SCI NP participants enrolled (I/C)**	**Sex (male/** **female)**	**Grade of ASIA**	**Age (Year) [Mean (SD)]** **Or (Median [IQR / Range)] (I/C)**	**NP duration (Month) [Mean (SD)] or (Median [IQR/Range)] (I/C)**	**Follow-up time (Weeks)**	**Pain evaluation tools**	**Overall assessment of risk of bias**	**Evaluation tools of mental or sleep-related symptom relief**
Ref. (18)	Nct 2012	UK	cannabinoids & placebo	116 (56 / 60)	91 / 25	NA	48.7 (12.97) / 47.6 (12.69)	NA	7	NRS	high	NRS
Ref. (19)	Cardenas 2013	USA	pregabalin & placebo	219 (111 / 108)	176 / 43	NA	46.1 (12.7) / 45.6 (13.8)	≥3	16	NRS	high	MOS-SS
Ref. (20)	Agarwal 2017	India	amitriptyline & lamotrigine	147 (74 / 73)	136 / 11	A-D	29.6 (18–40)	NA	3	SFMPQ2	high	NA
Ref. (21)	Amr 2010	Egypt	ketamine & gabapentin	40 (20 / 20)	33 / 7	A-D	48.6 (10.1) / 48.7 (9.7)	8 (6–17) / 9 (7- 18)	4	VAS (100)	moderate	NA
Ref. (22)	Amr 2011	Egypt	ketamine & gabapentin	40 (20 / 20)	33 / 7	A-D	48.6 (10.1) / 48.7 (9.7)	8 (6–17) / 9 (7–18)	8	VAS (100)	moderate	NA
Ref. (23)	Andresen 2016	Denmark	cannabinoids & placebo	73 (36 / 37)	54 / 19	A-D	58.6 (11.3)/54.1 (11.7)	≥3	12	NRS	moderate	NA
Ref. (24)	Salinas 2012	Colombia	carbamazepine & placebo	46 (24 / 22)	42 / 4	A-D	45.6 (18–70)	NA	24	VAS (100)	high	SF-36 Scale
Ref. (25)	Siddall 2006	Australia	pregabalin & placebo	137 (70 / 67)	114 / 23	A-D	50.3 (23–78) / 49.8 (21–80)	9.9 (7.7) / 10.4 (9.8)	12	NRS	high	MOS-sleep scale
Ref. (26)	Tai 2016	USA	gabapentin & placebo	14 (7 / 7)	12 / 2	A-D	35.9 (8.96) / 35.9 (8.96)	42.5 (88.8) / 42.5 (88.8)	10	NPS	moderate	NA
Ref. (27)	Vranken 2008	Netherlands	pregabalin & placebo	40 (20 / 20)	21 / 19	A-D	54.2 (9.4) / 54.7 (9.7)	≥6	4	VAS (10)	moderate	EQ-5D PDI
Ref. (28)	Vranken 2011	Netherlands	duloxetine & placebo	36 (18 / 18)	NA	A-D	NA	NA	8	VAS (10)	high	EQ-5D PDI
Ref. (29)	Yilmaz 2015	Turkey	pregabalin & gabapentin	30 (15 / 15)	25 / 5	A-D	32.93 (11.87)	31.48 (11.08)	18	VAS (10)	moderate	PDI
Ref. (30)	Chun2019	USA	BTX-A & placebo	8 (5 / 3)	6 / 2	A	45 (32–61)	≥1	12	NPRS	moderate	Pain of sleep
Ref. (31)	Finnerup 2009	Denmark	levetiracetam & placebo	36 (18 / 18)	29 / 7	A-D	51 (11.2)	≥3	5	NRS	high	Sleep interference
Ref. (32)	Han 2016	Korea	BTX-A & placebo	40 (20 / 20)	29 / 11	A-D	53.1 (9.1) / 48.9(14.2)	≥3	8	VAS (100)	high	SF-MPQ scores
Ref. (33)	Kaydok 2014	Turkey	gabapentin & pregabalin	28 (14 / 14)	21 / 7	A-D	42.8 (12.3)	29.3 (25.8)	4	VAS (100)	moderate	NPS
Ref. (34)	Levendoglu 2004	Turkey	gabapentin & placebo	20 (10 / 10)	13 / 7	NA	35.9 (9.8)	15.8 (9.0)	8	VAS (100)	moderate	NPS
Ref. (35)	Norrbrink 2009	Sweden	tramadol & placebo	35 (23 / 12)	28 / 7	NA	51.3 (10.8)	≥6	4	MPI scales	high	HAD
Ref. (36)	Rintala 2007	USA	amitriptyline & gabapentin & placebo	79 (28 / 26 / 25)	NA	A-D	42.6 (12.6)	>6	8	VAS / NRS	moderate	NA
Ref. (37)	Rintala2010	USA	cannabinoids & placebo	14 (7 / 7)	NA	A-D	50 (8.3)	>6	4	NRS	moderate	NA

*SCI, spinal cord injury; NP, neuropathic pain; ASIA, American Spinal Injury Association; SD, standard deviation; IQR, inter-quartile range; VAS, visual analog scale; NRS, numerical rating scale; SFMPQ2, short-form McGill pain questionnaire-2; NPS, numerical pain scale; NPRS, numerical pain rating scale; MPI, multidimensional pain inventory; MOS-SS, medical outcomes study sleep scale; SF-36 Scale, the short-form 36 item health survey questionnaire; EQ-5D, EuroQol five dimensions questionnaire; PDI, pain disability index; HAD, hospital anxiety, and depression*.

### Study Quality Control

Cochrane Handbook quality evaluation criteria were used by two researchers for independent evaluations of the publications. Criteria were as follows: random sequence generation, allocation hiding, participant and researcher blinding, result in evaluator blinding, outcome indicator integrity, selective reporting, and other sources of bias. Each item was scored as low, unclear, or high risk of bias.

### Statistical Analysis

Bayesian framework network meta-analysis was performed using OpenBUGS [version 3.2.3; (38)] and R [version 3.6.2; (39)] for repetitive proof. Standardized mean differences (SMDs) for continuous outcomes and summary odds ratios (ORs) for dichotomous outcomes were estimated using both pairwise and network meta-analysis and 95% credible interval (CrI) calculated. Group-level data were used for network meta-analysis with the binomial likelihood for dichotomous outcomes and normal likelihood for continuous outcomes. Study effect sizes were synthesized using a random-effects network meta-analysis model.

Three Markov chains were used to set the initial value, and the number of iterations for the first model update was set to 50,000 and for the continuous update to 100,000. The first 50,000 annealing times were discarded to eliminate the effect of initial values and sampling started from 50,001 times. The data fitting effect is shown in Supplementary Figure 1. Primary outcome pain scores and adverse events were used as outcome indicators to draw an evidence network map. In the case of a closed-loop, inconsistencies between direct and indirect comparisons were assessed through node splitting, and a *p* < 0.05 demonstrated that the inconsistency did not occur by chance (40). The inconsistency factor (IF) is close to zero with the 95% CrI included of zero, indicating good consistency between direct and indirect evidence (41). Surfaces under the cumulative ranking curve (SUCRA) and mean ranks were used to rank treatments with respect to each outcome (42). The area under the cumulative ranking probability map allowed assessment of each intervention as the potential best treatment. Higher SUCRA scores demonstrated better efficacy or safety. Statistical evaluation of inconsistent network graphs and results figures were processed using the network and network graphs packages of Stata (MP 14.2).

The current study was registered in the International Platform of Registered Systematic Review and Meta-Analysis Protocols (INPLASY), No.: INPLASY202070061, 10.37766/inplasy2020.7.0061. The original protocol is presented in the Supplementary Protocol.

## Results

**Figure 1:**
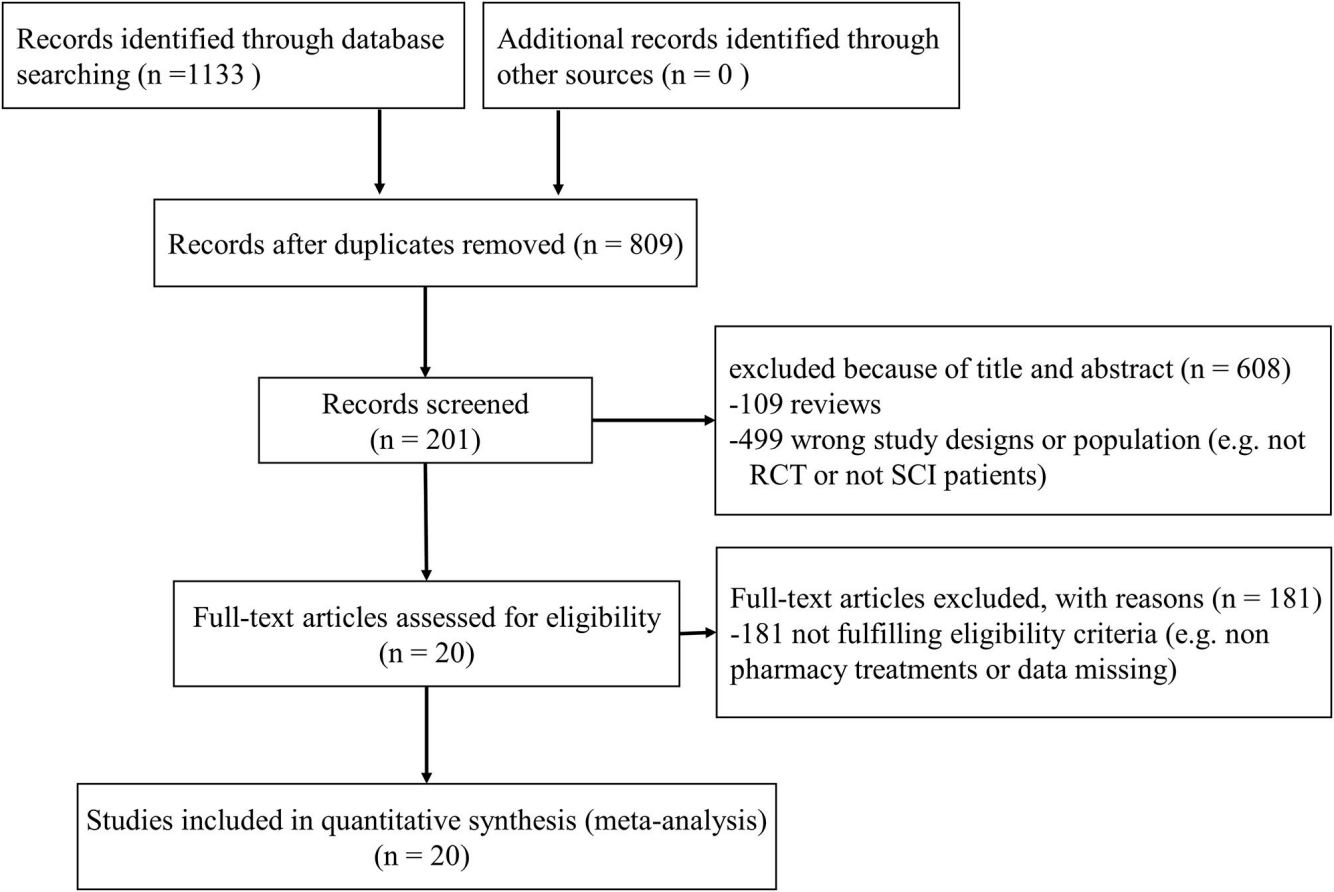
A study selection process.

**Figure 2:**
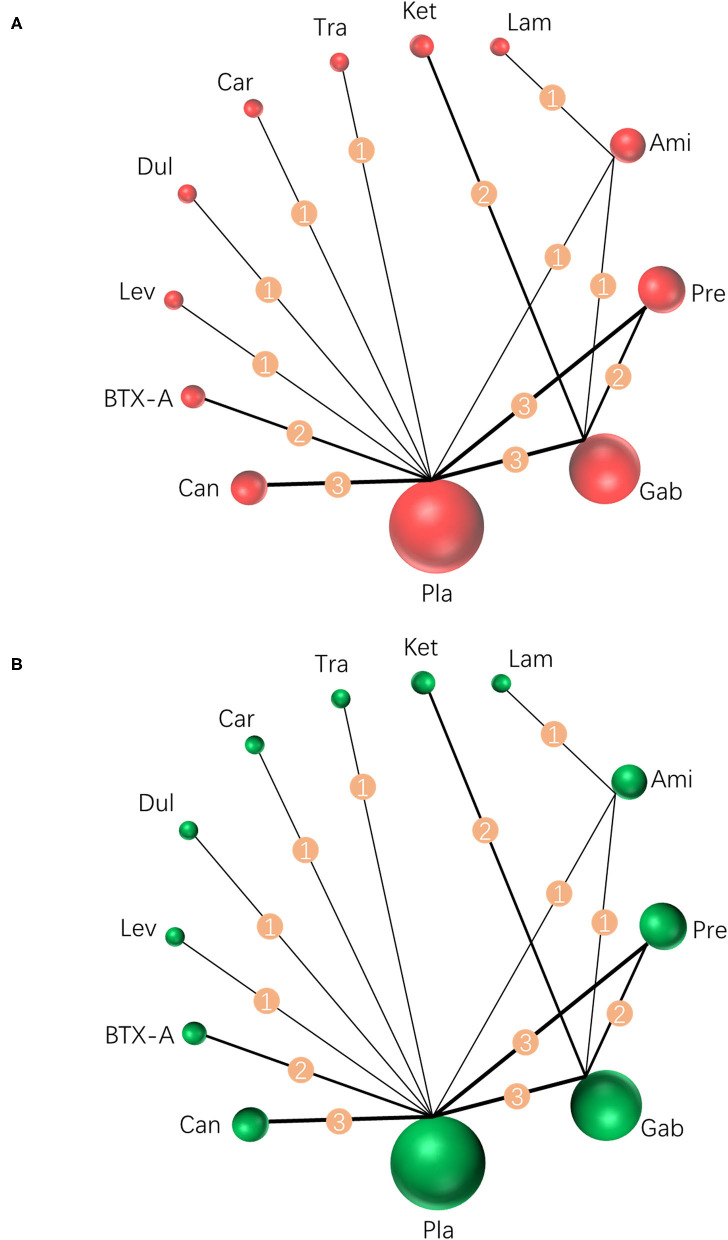
Network meta-analysis of eligible comparisons for primary outcomes. **(A)** Pain relief for around 4 weeks, *n* = 1,207. **(B)** Any adverse events, *n* = 1,232. Width of the lines is proportional to the number of trials, comparing every pair of treatments (numbers on the lines). Size of every circle is proportional to the number of randomly assigned participants (sample size). BTX-A, botulinum toxin-A; Ket, ketamine; Ami, amitriptyline; Lam, lamotrigine; Pre, pregabalin; Dul, duloxetine; Gab, gabapentin; Tra, tramadol; Lev, levetiracetam; Car, carbamazepine; Can, cannabinoids; Pla, placebo.

**Figure 3:**
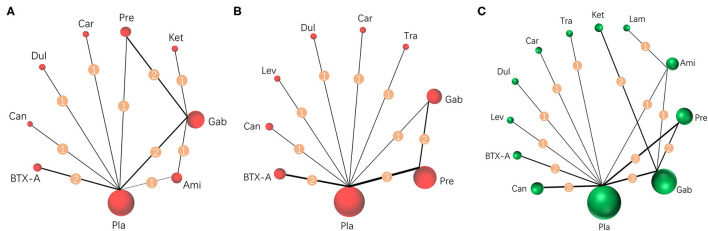
Network meta-analysis of eligible comparisons for secondary outcomes. **(A)** Pain relief more than 8 weeks, *n* = 615. **(B)** Mental or sleep-related symptom relief, *n* = 825. **(C)** Serious adverse events, *n* = 1,232. Width of the lines is proportional to the number of trials, comparing every pair of treatments for secondary outcomes (numbers on the lines). Size of every circle is proportional to the number of randomly assigned participants (sample size). BTX-A, botulinum toxin-A; Ket, ketamine; Ami, amitriptyline; Lam, lamotrigine; Pre, pregabalin; Dul, duloxetine; Gab, gabapentin; Tra, tramadol; Lev, levetiracetam; Car, carbamazepine; Can, cannabinoids; Pla, placebo.

Results were reported according to the preferred reporting items for systematic reviews and meta-analyses (PRISMA) extension statement (Supplementary Material) for network meta-analyses (43). A total of 1,133 citations were identified with 201 potentially eligible full texts. A flow chart outlining the selection process is shown in Figure 1. Between 1963 and 2021, there were 20 RCTs (1,198 patients), comparing 11 drugs or placebos [Table 1; (18–37)]. The mean number of participants was 60 ± 55. A total of 622 participants were randomly administered drugs, whereas 576 received placebos. Most patients suffered from moderate to severe pain. Drug-based (pain relief medication) and non-drug therapy (physical and psychological educational therapies) were included. Nineteen out of 20 trials (95%) were double-arm studies, and the remaining one (5%) was a three-arm study. Fourteen (70%) compared drugs with placebo and 5 (25%) compared two different drugs. Network estimates of the main outcomes were based on moderate to high certainty of evidence (Supplementary Figures 2, 3). Network analysis of primary and secondary outcomes conformed with the consistency model (Supplementary Figures 4, 5). All trials assessed during the current study involved the following 11 drugs: BTX-A, ketamine, amitriptyline, lamotrigine, pregabalin, duloxetine, gabapentin, tramadol, levetiracetam, carbamazepine, and cannabinoids. A network of eligible comparisons is presented for primary outcomes in Figure 2 and for secondary outcomes in Figure 3. The circle area represents the number of studies included in each treatment group, and the line width represents the number of comparative trials. All studies were RCTs, assessing drug action vs. a placebo, except for those involving ketamine and lamotrigine.

**Figure 4:**
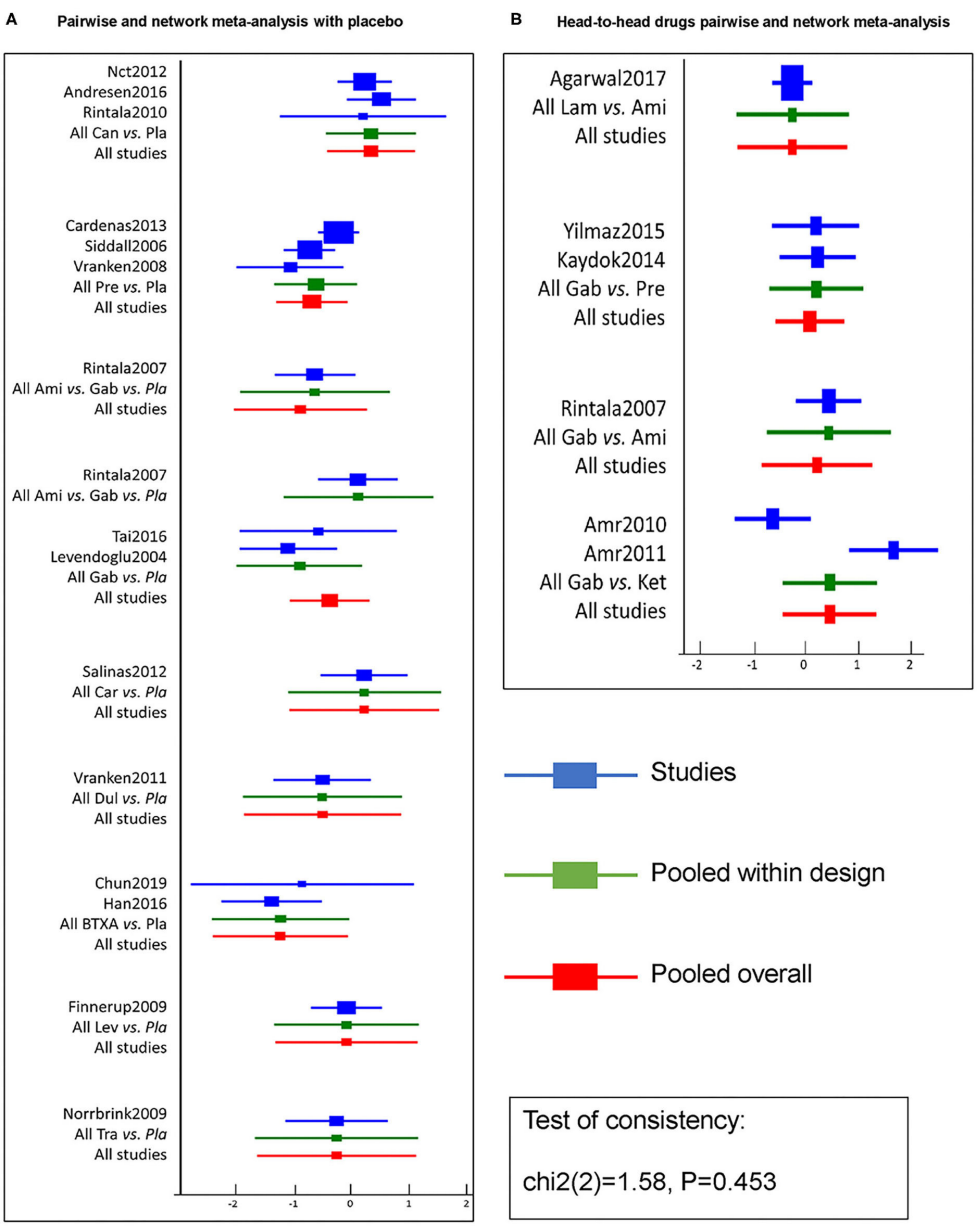
Efficacy pairwise and network meta-analysis of 11 drugs and placebo from 20 RCTs. A forest plot **(A)** shows pairwise and network meta-analysis of different drugs on the target of neuropathic pain relief after SCI as compared with placebo. And the forest plot **(B)** shows pairwise and network meta-analysis among head-to-head drugs. The blue line in forest plots indicates the outcome analysis of corresponding studies, the green line refers to pooled within designed treatments, the red line means pooled overall. The test of consistency reveals all studies are according to the consistency model. BTX-A, botulinum toxin-A; Ket, ketamine; Ami, amitriptyline; Lam, lamotrigine; Pre, pregabalin; Dul, duloxetine; Gab, gabapentin; Tra, tramadol; Lev, levetiracetam; Car, carbamazepine; Can, cannabinoids; Pla, placebo.

**Figure 5:**
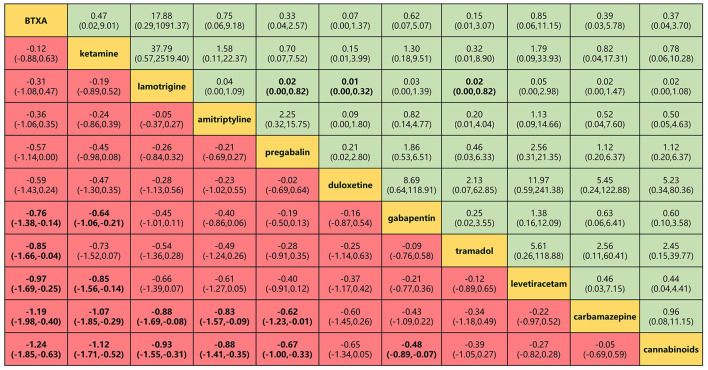
Head-to-head comparisons for efficacy and safety (primary outcomes) of 11 drugs. Drugs are reported in efficacy order sorted from top left to bottom right. Data are SMD (95% CrI) and OR (95% CrI) in the column-defining treatment compared with the row-defining treatment. For efficacy (pain relief for around 4 weeks), SMD lower than 0 favors the column-defining treatment. For safety (adverse events), OR lower than 1 favors near the top left one. To obtain OR for comparisons in the opposite direction, reciprocals should be taken. Significant results are in bold. SMD, standard mean deviation; OR, odds ratio; CrI, credible interval; BTXA, botulinum toxin-A.

**Figure 6:**
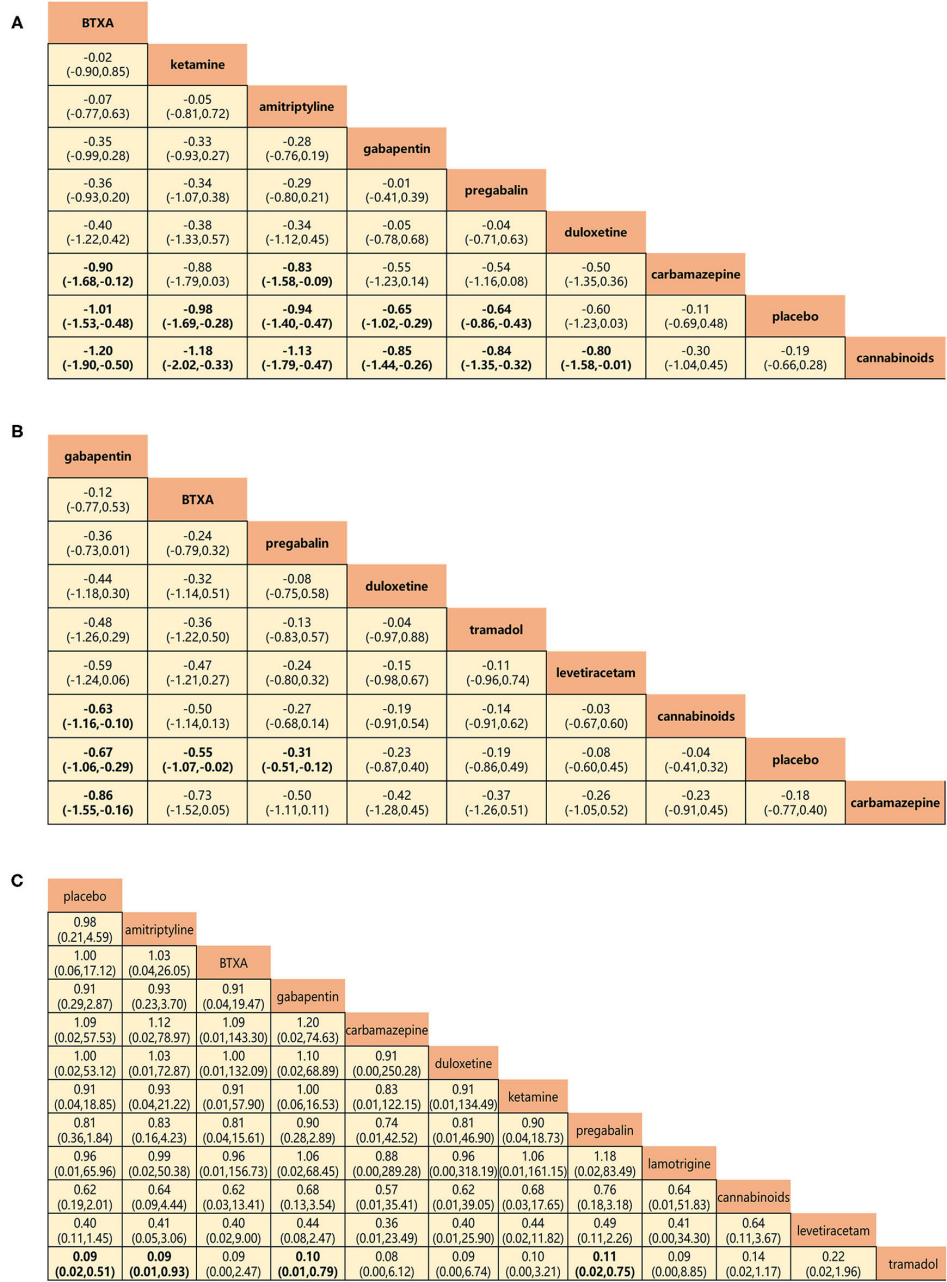
Head-to-head comparisons for secondary outcomes of drugs and placebo. **(A)** Pain relief more than 8 weeks (8 drugs). **(B)** Mental or sleep-related symptom relief (8 drugs). **(C)** Serious adverse events (11 drugs). Drugs are reported in efficacy and safety order sorted from top left to bottom right. Data are SMDs (95% CrI) and ORs (95% CrI) in the column-defining treatment compared with the row-defining treatment. For A and B, SMDs lower than 0 favor the column-defining treatment. For C, ORs lower than 1 favor near the top left one. Significant results are in bold. SMD, standard mean deviation; OR, odds ratio; CrI, credible interval; BTXA, botulinum toxin-A.

Pairwise efficacy and network meta-analysis of 11 drugs with respect to placebos from 20 RCTs are visualized as the forest plot shown in Figure 4. Pairwise and network meta-analysis of other outcomes are presented in Supplementary Figure 6. Inconsistent test node split analysis for primary and secondary outcomes was also performed, and a *p* < 0.05 or DIC (inconsistency) minus DIC (consistency) of <5 was taken to indicate significant inconsistency. Results gave *p*-values of over 0.05 (Figure 4 and Supplementary Figure 6), indicating consistency between primary and secondary outcomes, and direct and indirect results. Detailed results of a pair of meta-analyses of primary and secondary outcomes of 11 drugs are presented in Figures 5, 6, respectively.

BTX-A, ketamine, amitriptyline, lamotrigine, pregabalin, and gabapentin were shown to be the most effective of the drugs tested for the primary outcomes of efficacy and safety (Figure 5). No significant differences were found among the 11 drugs in terms of adverse event rates. BTX-A was ranked as the most effective drug for pain relief at 4 weeks of follow-up. SMDs for BTX-A with respect to other drugs (gabapentin, tramadol, levetiracetam, carbamazepine, and cannabinoids) ranged from −0.76 to −0.24). Ketamine was also among the more effective drugs with SMDs compared with others (gabapentin, levetiracetam, carbamazepine, and cannabinoids), ranging from −0.44 to −1.12. No significant differences were found among lamotrigine, amitriptyline, and gabapentin, but their efficacy was higher than that of other drugs (carbamazepine and cannabinoids) with SMDs, ranging from −0.88 to −2.81. However, tramadol, levetiracetam, and cannabinoid did not produce significantly different outcomes when compared with one another or with a placebo. Lamotrigine had the best safety profile with respect to adverse events of the primary outcome when compared to pregabalin 0.02 (0.001–0.82), duloxetine 0.01 (0.001–0.32), and tramadol 0.02 (0.001–0.82).

Pain relief at 8-week follow-up and relief of mental or sleep-related symptoms represented the secondary outcome of efficacy, and network analysis results were in agreement with those for the primary outcome. BTX-A, ketamine, amitriptyline, gabapentin, and pregabalin showed greater long-term efficacy of pain relief (SMDs: −0.90 to −1.20; Figure 6A). Gabapentin, BTX-A, and pregabalin were more successful in relieving mental or sleep-related symptoms with SMDs, ranging from −0.63 to −0.86 (Figure 6B). The incidence of serious adverse events represented the secondary outcome for the assessment of safety (Figure 6C). No significant differences were found for any drug, with the exception of tramadol, which triggered more serious adverse events than any other (ORs: 0.09–0.11).

**Figure 7:**
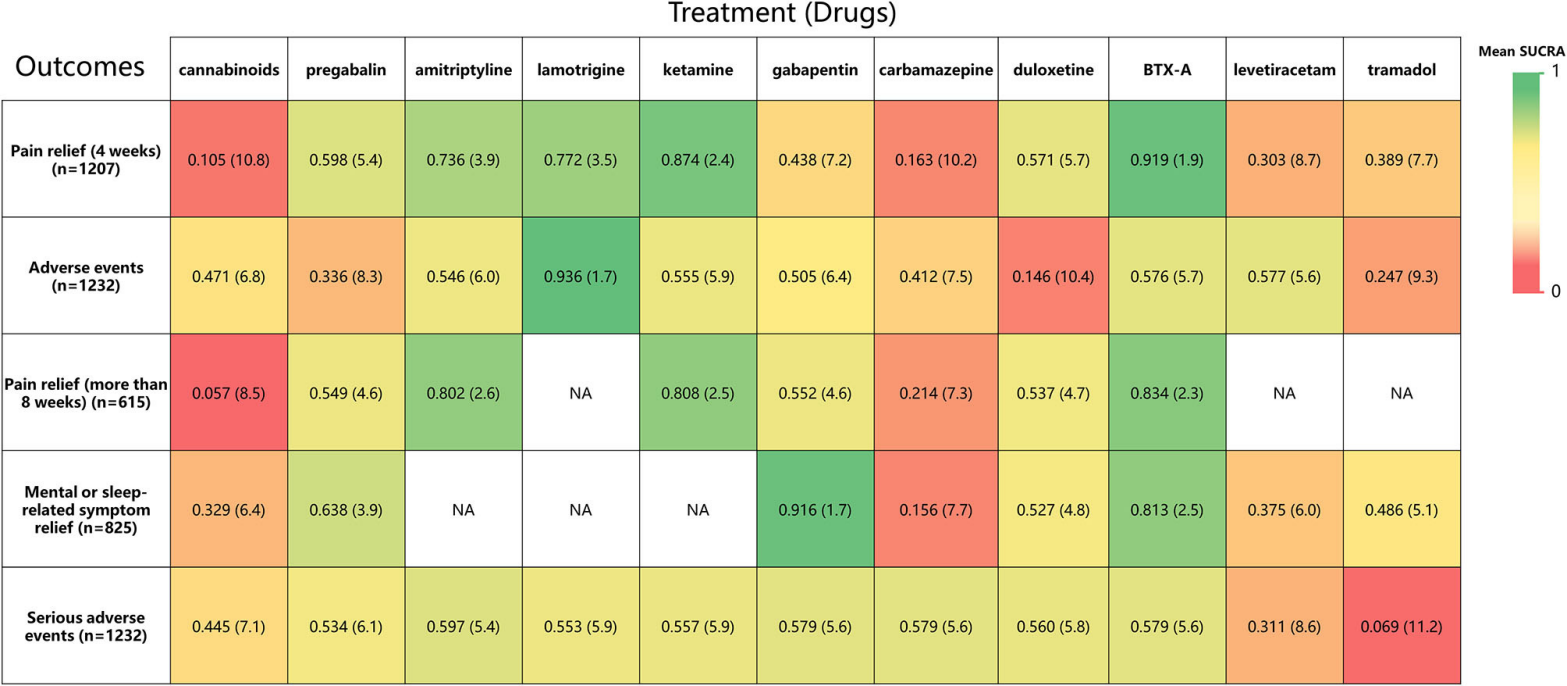
A heat map of 11 treatment drugs studied in patients with SCI with neuropathic pain for 5 outcomes. Each column represents a treatment drug, and each row represents an outcome. For each outcome (Column 1), the number of studies included in the analysis is presented in parentheses. SUCRA, surface under the cumulative ranking curve. Each box is colored according to the mean SUCRA value of the corresponding treatment and outcome. The color scale consists of values that represent mean SUCRA, which ranges from 0 (red, indicating a treatment is always last) to 1 (green, indicating a treatment is always first). Uncolored boxes labeled NA (data not available) show that the underlying treatment was not included for that particular outcome. The values in each box represent the mean SUCRA value and mean rank of the corresponding drug and outcome.

Rankings for all 11 drugs were based on SUCRA values for primary and secondary outcomes. Combined with network meta-analysis results on the efficacy of pain relief at 4-week follow-up (Supplementary Figure 6), BTX-A was ranked first, followed by ketamine, amitriptyline, lamotrigine, pregabalin, duloxetine, gabapentin, tramadol, levetiracetam, carbamazepine, and cannabinoids. SUCRA safety rankings based on adverse event incidence (Supplementary Figure 7) were as follows: lamotrigine, BTX-A, levetiracetam, amitriptyline, gabapentin, ketamine, carbamazepine, cannabinoids, pregabalin, tramadol, and duloxetine. SUCRA rankings of secondary outcomes were very similar to those for primary outcomes, except gabapentin was the most effective in mental or sleep-related symptom relief and tramadol had the worst safety profile (Supplementary Figures 8–11). A net funnel for primary outcomes is shown in Supplementary Figures 12, 13 and indicates that publication bias was under control. A heat map depicting the hierarchy of all 11 drugs according to mean SUCRA values across 5 primary and secondary outcomes is shown in Figure 7. In summary, the overall rankings are BTX-A, gabapentin, ketamine, amitriptyline, pregabalin, duloxetine, lamotrigine, levetiracetam, tramadol, carbamazepine, and cannabinoids. However, due to the paucity of data, quantitative synthesis was not performed on the remaining *priori*-defined outcomes.

## Discussion

Davis and Martin (44) have vividly delineated SCI-related NP: hot, burning pain would be replaced by severe crushing pressure, vise-like, pinching sensations, streams of fire running down the legs into the feet and out of the toes or a pain produced by the pressure of a knife being buried in the tissue, twisted around rapidly and simultaneously withdrawn. In stark contrast to the profound effectiveness of opioids in relieving nociceptive pain, there is, unfortunately, no similar panacea for the treatment of NP (45). To advance the evidence-based approach to drug management of SCI-related NP, the current study conducted a Bayesian network analysis and managed to make a discussion per drug based on the results of network analysis and etiology and/or pharmacology of NP as follows.

### BTX-A

BTX-A is a complex proteinaceous neurotoxin with proteolytic activity, which affects both the synaptic and auxiliary proteins involved in the release of vesicle neurotransmitters (eg., acetylcholine (Ach)) and/or other local neuropeptides [e.g., calcitonin gene-related peptides (CGRP) and substance P] (46). However, BTX-A's dual mechanisms of action are controversial (47). Favre-Guilmard et al. (48) and Park and Chung (49) have demonstrated that BTX-A inhibits the secretion of substance P and CGRP from dorsal root ganglion (DRG), restrains the expression of TRPV1 and P_2_X_3_, and induces a central effect through retrograde axonal transport. Those reports particularly emphasize BTX-A's potential character in reducing NP. Although Finnerup et al. (50) have recommended BTX-A as a fourth-line treatment, there is still increasing interest in its use for the management of NP (47, 51, 52). Therefore, oral or injected administration of BTX-A has been shown to induce analgesia in neuro-related or neurogenic pain (53–55). Similarly, BTX-A (200 U) was administered subcutaneously during two RCTs (32, 56) referred to in the current study and alleviated intractable post-SCI NP as the previous report (57). More importantly, guidelines published by the American Academy of Neurology recommend the use of BTX-A, illustrating its effectiveness (Level A) in SCI-induced NP (58). The current study provides further evidence to support the efficacy of BTX-A *via* network meta-analysis within the Bayesian evidence framework. However, optimal routes of BTX-A administration and dosage remain unclear. The current findings may further knowledge of pain mechanisms and give insights into the potential for novel-engineered toxins that specifically target pain neurotransmitters.

### Ketamine

The α2δ-1-bound NMDA receptors, in particular, are involved in the pathology of NP and present a potential target for NP therapy (59–61). Luo et al. (62) reported that repeated activation of presynaptic fibers leads to long-term potentiation (LTP), which represents a vital mechanism in producing central pain. In combination with activation of NMDA receptors, such algogenic mechanisms probably relate to reinforcing central sensitization (45, 63). Coincidentally, ketamine could antagonize NMDA receptor-mediated central sensitization by blocking harm signal inputs (64) and had been considered safe and effective in reducing chronic pain (65–67). Also, Amr (21, 22) studied the efficacy of long-term intravenous drip and epidural injection of ketamine for post-SCI NP. Despite the safety and effectiveness of the drug, its analgesic effects wore off 2 weeks after discontinuation of the infusion, although the epidural route produced more prolonged efficacy (1 month). The current study found ketamine suitable for the management of post-SCI NP, as it was effective with few adverse events (Figure 5 and Supplementary Figures 8, 11). Nevertheless, the necessity of administration *via* intravenous drip or epidural injection reduces its convenience compared with other drugs.

### Gabapentinoids

Gabapentin and pregabalin are gabapentinoids that target the α2δ-1 auxiliary subunit of voltage-gated Ca^2+^ channels. Although structurally similar to GABA, gabapentin does not affect the uptake, synthesis, or metabolism of GABA (68) nor do either gabapentin or pregabalin bind to GABAA or GABAB receptors (69–71). Hence, gabapentin is commonly recommended as immediate therapy for the management of post-SCI NP (72, 73). Besides, pregabalin as a new generation of gabapentinoid is the only drug approved by the US Food and Drug Administration (FDA) for the treatment of post-SCI NP. Moreover, gabapentin and pregabalin were extensively compared in the systematic review and meta-analysis by Davari et al. (74). No significant difference between the two drugs was reported in terms of change of a pain score and safety. The current network meta-analysis produced findings consistent with those of previous studies (Figure 5). Although pregabalin resulted in more adverse events (Supplementary Figure 8), these tended to be minor and well tolerated. More importantly, gabapentin proved to be particularly valuable for its relief of mental or sleep-related symptoms (Figure 6). The current findings reinforce the view that gabapentinoids are suitable first-line treatment options for NP. Novel information about their mechanism of action may lead to improved treatment management, and, thus, in combination with the previous findings (45, 75), we also support the perspective that combining gabapentinoids with other active drugs might be expected to improve overall therapeutic effectiveness in post-SCI NP.

### Serotonin and Noradrenaline

Amitriptyline and duloxetine target serotonin or 5-hydroxytryptamine (5-HT) and noradrenaline (NA) uptake systems to have an impact on the nociceptive regulatory circuit (76). Amitriptyline is a tricyclic antidepressant with analgesic action in neuropathy and deafferentation-induced autotomy (77). It is the first-line drug of choice for treating SCI-related pain (78), although Rintala et al. (36) conducted an RCT that was double-blind and triple-cross over 8 weeks and found that high-dose amitriptyline (150 mg/day) had moderate efficacy, compared with gabapentin or a placebo, but only in a subgroup of patients with depressive symptoms. For another, the clinical efficacy of duloxetine depends on dual reuptake inhibition at the condition-specific site within the central nervous system (79). Vranken et al. (28) found no improvement in pain intensity in patients with SCI during an 8-week double-blinded, placebo-controlled study, employing a flexible-dose regimen of duloxetine. By contrast, Ziegler et al. (80) found duloxetine to be effective for the treatment of diabetic peripheral neuropathic pain (DPNP) in a placebo-controlled study. The current network analysis ranked amitriptyline highly for pain relief, both at 4-week and long-term follow-up (Figures 5, 6), while duloxetine produced many more adverse events (Figure 6 and Supplementary Figure 8). Such conclusions are consistent with those of Finnerup et al. (50) whose meta-analysis identified amitriptyline as a first-line drug. However, duloxetine has utility as a first-line treatment option for DPNP, but its suitability in relation to post-SCI NP remains uncertain.

### Voltage-Gated Na^+^ Channels

Lamotrigine and carbamazepine act on voltage-sensitive sodium channels, stabilize neuronal membranes, and inhibit the pathological release of excitatory amino acid transmitters, such as glutamate, that are mediated by sodium influx. Agarwal and Joshi (20) found that lamotrigine is associated with fewer adverse events and should be the first-choice drug for NP. This proposes lamotrigine a favorable drug for NP treatment as in previous reports (50, 75). Similarly, carbamazepine presents an FDA-approved drug for epilepsy, bipolar disorder, and NP treatment (81, 82). A double-blind, placebo-controlled RCT plus parallel-group study conducted by Salinas et al. (24) found that early carbamazepine did not reduce overall NP incidence or intensity in the long run. Besides, many studies have indicated the suitability of the anticonvulsant carbamazepine for the treatment of trigeminal neuralgia (83–85). The current findings that lamotrigine was markedly more effective for the treatment of post-SCI NP than that of carbamazepine were consistent with those of previous studies (45, 50, 75). Moreover, lamotrigine produces fewer adverse events. Carbamazepine is much less effective for management of SCI-related NP than for trigeminal neuralgia.

### Tramadol and Levetiracetam

Tramadol and levetiracetam, an opioid analgesic and anticonvulsant, respectively, were generally reported to be minimally effective in the treatment of SCI-related NP (31, 35). On the one hand, tramadol produced more adverse effects than many other drugs, in agreement with the findings of Norrbrink and Lundeberg (35) and Finnerup et al. (86). These authors stressed the adjuvant role of tramadol and other opioids, indicating that gabapentin, pregabalin, serotonin, and norepinephrine reuptake inhibitors should first be tried for NP. Besides, numerous studies have recommended opioid titration with low initial and slow individual doses to mitigate adverse events. On the other hand, Finnerup et al. (31) indicated that levetiracetam in doses titrated up to 3,000 mg had no analgesic or other benefits in patients with post-SCI NP during a double-blind, placebo-controlled, crossover, multicenter RCT. The reason for the lack of efficacy of levetiracetam is unknown, but doses used may be insufficient to affect *SV2A* and have an impact on NP. Lastly, the current findings are to contra-indicate the use of tramadol and levetiracetam for SCI-related NP due to poor efficacy and high risk of adverse events (Figures 5–7).

### Cannabinoids

Cannabinoids activate the CB1 and/or CB2 receptors, but any potential role in the treatment of SCI-related NP remains unclear (18). Cannabinoids produce analgesic effects due to actions in and around the brain and spinal cord, but use is limited by side effects and concerns over long-term risks (50, 87, 88). The current network meta-analysis suggested little effect of cannabinoids compared with a placebo, producing a SUCRA ranking in the last place. Thus, cannabinoids may not be appropriate for the treatment of post-SCI NP, mainly due to potential abuse, diversion, and long-term mental health risks, especially in susceptible populations (45, 50).

### Recommendation and Insight

To the best of our knowledge, the current comprehensive network meta-analysis is the first to address the pharmacological treatment of NP after SCI. Our findings are different from previous meta-analyses, which were limited to a single drug, a pair of drugs, or a class of drugs. Bayesian inference provides a statistical framework for the integration of current data, prior knowledge, and reasonable assumptions about the system to generate probability distributions (89). Hence, a Bayesian framework was used to implement the network meta-analysis and summarize the two primary and three secondary outcomes of drug efficacy and safety. In summary, gabapentin, BTX-A, amitriptyline, ketamine, and lamotrigine had relatively high pain relief efficacy with fewer adverse events than other drugs, which support them as first-line therapy for post-SCI NP. Pregabalin and duloxetine had some degree of efficacy in NP relief, and they are proposed as second-line treatment because their safety should be carefully considered. Tramadol, levetiracetam, carbamazepine, and cannabinoids had lower efficacy and worse safety profiles than other drugs, making them less favorable in treating post-SCI NP. Although all drugs were accompanied by adverse effects to varying degrees, most of them were acceptable and well tolerated. Undoubtedly, head-to-head studies were prioritized, and the certainty of the retrieved evidence was confirmed within this finding. Totally, few differences were found among the scrutinized drugs, and differences in efficacy and safety were minimal. Anyway, the patient's individual perception of the disease and its symptoms proximately results in variable therapeutic efficacy and acceptability of side effects. Therefore, further multicenter, large sample RCTs, mechanistic and data-mining studies for each drug are required to extend evaluations and reduce the bias of significant innovation.

## Study Limitations

Meta-analyses have some limitations due to the reliance on statistical assumptions and data-based simulation, and the rigor of the research methodology of individual studies is a potential confounding factor. We acknowledge some limitations to the present study. Firstly, some RCTs included in the current analysis had fewer than 50 participants. Secondly, SCI-related NP was generalized and not divided into subgroups (NP at the injured level and below the injured level). Thirdly, only drug efficacy and safety were compared without taking into account dosage and frequency or availability and cost – neither was the drug delivery route scrutinized. Fourthly, some studies included in the present meta-analysis were funded by drug companies, which may carry a risk of bias.

## Conclusion

The management of post-SCI NP was analyzed within the framework of Bayesian network analysis to demonstrate that the efficacy and safety of BTX-A make it a suitable drug choice. Ketamine is also beneficial but has to be administered *via* intravenous drip or epidural injection. Gabapentin and amitriptyline remain appropriate first-line treatments for NP. Lamotrigine, pregabalin, and duloxetine have some utility as well. It is noteworthy that lamotrigine and gabapentin have fewer side effects and help relieve mental or sleep-related symptoms. Tramadol, levetiracetam, carbamazepine, and cannabinoids have poor safety or efficacy and are not recommended for SCI-related NP treatment, unless as adjuvants.

## Data Availability Statement

The original contributions presented in the study are included in the article/Supplementary Material, further inquiries can be directed to the corresponding authors.

## Author Contributions

H-QL and Z-HC conceptualized and designed the study, analyzed the data, and drafted and revised the manuscript. LH and FF designed the data collection instruments, collected data, and carried out the initial analyses. C-GW and S-JC conceptualized and designed the study, coordinated, supervised data collection, and critically reviewed the manuscript for important intellectual content. P-GX and L-MR contributed greatly to the design, correction, and supplement of the study. All authors approved the final manuscript as submitted and agreed to be accountable for all aspects of the work.

## Funding

This study was supported by the National Key Research and Development Program of China (2017YFA0105403). The funding source had no involvement in study design, data collection, analysis, interpretation of data, writing of the report, or the decision to submit the article for publication.

## Conflict of Interest

The authors declare that the research was conducted in the absence of any commercial or financial relationships that could be construed as a potential conflict of interest.

## Publisher's Note

All claims expressed in this article are solely those of the authors and do not necessarily represent those of their affiliated organizations, or those of the publisher, the editors and the reviewers. Any product that may be evaluated in this article, or claim that may be made by its manufacturer, is not guaranteed or endorsed by the publisher.

## References

[1] Lee BB Cripps RA Fitzharris M Wing PC. The global map for traumatic spinal cord injury epidemiology: update 2011, global incidence rate. Spinal Cord. (2014) 52:110–6. 10.1038/sc.2012.158 23439068

[2] Furlan JC Sakakibara BM Miller WC Krassioukov AV. Global incidence and prevalence of traumatic spinal cord injury. Can J Neurol Sci. (2013) 40:456–64. 10.1017/S0317167100014530 23786727

[3] Noristani HN Lonjon N Cardoso M Le Corre M Chan-Seng E Captier G . Correlation of *in vivo* and *ex vivo* (1)H-MRI with histology in two severities of mouse spinal cord injury. Front Neuroanat. (2015) 9:24. 10.3389/fnana.2015.00024 25798092 PMC4350395

[4] Siddall PJ McClelland JM Rutkowski SB Cousins MJ. A longitudinal study of the prevalence and characteristics of pain in the first 5 years following spinal cord injury. Pain. (2003) 103:249–57. 10.1016/S0304-3959(02)00452-9 12791431

[5] Widerström-Noga EG Turk DC. Types and effectiveness of treatments used by people with chronic pain associated with spinal cord injuries: influence of pain and psychosocial characteristics. Spinal Cord. (2003) 41:600–9. 10.1038/sj.sc.3101511 14569261

[6] Soler MD Saurí-Ruiz J Curcoll-Gallemí ML Benito-Penalva J Opisso-Salleras E Chamarro-Lusar A . Characteristics of chronic neuropathic pain and their relationship with psychological well-being in spinal cord injury patients. Rev Neurol. (2007) 44:3–9. 10.33588/rn.4401.2005800 17199222

[7] Soler MD Kumru H Pelayo R Vidal J Tormos JM Fregni F . Effectiveness of transcranial direct current stimulation and visual illusion on neuropathic pain in spinal cord injury. Brain. (2010) 133:2565–77. 10.1093/brain/awq184 20685806 PMC2929331

[8] Hendrich J Van Minh AT Heblich F Nieto-Rostro M Watschinger K Striessnig J . Pharmacological disruption of calcium channel trafficking by the alpha2delta ligand gabapentin. Proc Natl Acad Sci U S A. (2008) 105:3628–33. 10.1073/pnas.0708930105 18299583 PMC2265195

[9] Gu Y Huang LY. Gabapentin actions on N-methyl-D-aspartate receptor channels are protein kinase C-dependent. Pain. (2001) 93:85–92. 10.1016/S0304-3959(01)00297-4 11406342

[10] Marek GJ McDougle CJ Price LH Seiden LS. A comparison of trazodone and fluoxetine: implications for a serotonergic mechanism of antidepressant action. Psychopharmacology. (1992) 109:2–11. 10.1007/BF02245475 1365657

[11] Hohmann AG Herkenham M. Localization of central cannabinoid CB1 receptor messenger RNA in neuronal subpopulations of rat dorsal root ganglia: a double-label in situ hybridization study. Neuroscience. (1999) 90:923–31. 10.1016/S0306-4522(98)00524-7 10218792

[12] Wu J Zhao Z Zhu X Renn CL Dorsey SG Faden AI. Cell cycle inhibition limits development and maintenance of neuropathic pain following spinal cord injury. Pain. (2016) 157:488–503. 10.1097/j.pain.0000000000000393 26797506 PMC4881432

[13] Cardenas DD Jensen MP. Treatments for chronic pain in persons with spinal cord injury: a survey study. J Spinal Cord Med. (2006) 29:109–17. 10.1080/10790268.2006.11753864 16739554 PMC1864800

[14] Siontis GC Mavridis D Greenwood JP Coles B Nikolakopoulou A Jüni P . Outcomes of non-invasive diagnostic modalities for the detection of coronary artery disease: network meta-analysis of diagnostic randomised controlled trials. BMJ. (2018) 360:k504. 10.1136/bmj.k504 29467161 PMC5820645

[15] Lu G Ades AE. Combination of direct and indirect evidence in mixed treatment comparisons. Stat Med. (2004) 23:3105–24. 10.1002/sim.1875 15449338

[16] Cipriani A Furukawa TA Salanti G Chaimani A Atkinson LZ Ogawa Y . Comparative efficacy and acceptability of 21 antidepressant drugs for the acute treatment of adults with major depressive disorder: a systematic review and network meta-analysis. Lancet. (2018) 391:1357–66. 10.1016/S0140-6736(17)32802-7 29477251 PMC5889788

[17] Hróbjartsson A Thomsen AS Emanuelsson F Tendal B Hilden J Boutron I . Observer bias in randomized clinical trials with measurement scale outcomes: a systematic review of trials with both blinded and nonblinded assessors. CMAJ. (2013) 185:E201–11. 10.1503/cmaj.120744 23359047 PMC3589328

[18] Nct. A *Study of Cannabis Based Medicine Extracts and Placebo in Patients With Pain Due to Spinal Cord Injury*. (2012). Available online at: https://clinicaltrialsgov/show/NCT01606202 (accessed May 31, 2018).

[19] Cardenas DD Nieshoff EC Suda K Goto S Sanin L Kaneko T . A randomized trial of pregabalin in patients with neuropathic pain due to spinal cord injury. Neurology. (2013) 80:533–9. 10.1212/WNL.0b013e318281546b 23345639 PMC3589291

[20] Agarwal N Joshi M. Effectiveness of amitriptyline and lamotrigine in traumatic spinal cord injury-induced neuropathic pain: a randomized longitudinal comparative study. Spinal Cord. (2017) 55:126–30. 10.1038/sc.2016.123 27527240

[21] Amr YM. Multi-day low dose ketamine infusion as adjuvant to oral gabapentin in spinal cord injury related chronic pain: a prospective, randomized, double blind trial. Pain Physician. (2010) 13:245–9. 10.36076/ppj.2010/13/245 20495588

[22] Amr YM. Epidural ketamine in post spinal cord injury-related chronic pain. Anesth Essays Res. (2011) 5:83–6. 10.4103/0259-1162.84196 25885306 PMC4173366

[23] Andresen SR Bing J Hansen RM Biering-Sorensen F Johannesen IL Hagen EM . Ultramicronized palmitoylethanolamide in spinal cord injury neuropathic pain: a randomized, double-blind, placebo-controlled trial. Pain. (2016) 157:2097–103. 10.1097/j.pain.0000000000000623 27227691

[24] Salinas FA Lugo LH García HI. Efficacy of early treatment with carbamazepine in prevention of neuropathic pain in patients with spinal cord injury. Am J Phys Med Rehabil. (2012) 91:1020–7. 10.1097/PHM.0b013e3182643c85 22854901

[25] Siddall PJ Cousins MJ Otte A Griesing T Chambers R Murphy TK. Pregabalin in central neuropathic pain associated with spinal cord injury: a placebo-controlled trial. Neurology. (2006) 67:1792–800. 10.1212/01.wnl.0000244422.45278.ff 17130411

[26] Tai Q Kirshblum S Chen B Millis S Johnston M DeLisa JA. Gabapentin in the treatment of neuropathic pain after spinal cord injury: a prospective, randomized, double-blind, crossover trial. J Spinal Cord Med. (2002) 25:100–5. 10.1080/10790268.2002.11753609 12137213

[27] Vranken JH Dijkgraaf MG Kruis MR van der Vegt MH Hollmann MW Heesen M. Pregabalin in patients with central neuropathic pain: a randomized, double-blind, placebo-controlled trial of a flexible-dose regimen. Pain. (2008) 136:150–7. 10.1016/j.pain.2007.06.033 17703885

[28] Vranken JH Hollmann MW van der Vegt MH Kruis MR Heesen M Vos K . Duloxetine in patients with central neuropathic pain caused by spinal cord injury or stroke: a randomized, double-blind, placebo-controlled trial. Pain. (2011) 152:267–73. 10.1016/j.pain.2010.09.005 21078545

[29] Yilmaz B Yasar E Koroglu Omac O Goktepe AS Tan AK. Gabapentin vs. pregabalin for the treatment of neuropathic pain in patients with spinal cord injury: a crossover study. Türkiye Fiziksel Tip ve Rehabilitasyon Dergisi. (2015) 61:1–5. 10.5152/tftrd.2015.79069

[30] Chun A Levy I Yang A Delgado A Tsai CY Leung E . Treatment of at-level spinal cord injury pain with botulinum toxin A. Spinal Cord Ser Cases. (2019) 5:77. 10.1038/s41394-019-0221-9 31632735 PMC6786298

[31] Finnerup NB Grydehoj J Bing J Johannesen IL Biering-Sorensen F Sindrup SH . Levetiracetam in spinal cord injury pain: a randomized controlled trial. Spinal Cord. (2009) 47:861–7. 10.1038/sc.2009.55 19506571

[32] Han ZA Song DH Oh HM Chung ME. Botulinum toxin type A for neuropathic pain in patients with spinal cord injury. Ann Neurol. (2016) 79:569–78. 10.1002/ana.24605 26814620 PMC4825405

[33] Kaydok E Levendoglu F Ozerbil MO Karahan AY. Comparison of the efficacy of gabapentin and pregabalin for neuropathic pain in patients with spinal cord injury: a crossover study. Act. Med. Meditter. (2014) 30:1343–8.

[34] Levendoglu F Ögün CÖ Özerbil Ö Ögün TC Ugurlu H. Gabapentin is a first line drug for the treatment of neuropathic pain in spinal cord injury. Spine. (2004) 29:743–51. 10.1097/01.BRS.0000112068.16108.3A 15087796

[35] Norrbrink C Lundeberg T. Tramadol in neuropathic pain after spinal cord injury: a randomized, double-blind, placebo-controlled trial. Clin J Pain. (2009) 25:177–84. 10.1097/AJP.0b013e31818a744d 19333166

[36] Rintala DH Holmes SA Courtade D Fiess RN Tastard LV Loubser PG. Comparison of the effectiveness of amitriptyline and gabapentin on chronic neuropathic pain in persons with spinal cord injury. Arch Phys Med Rehabil. (2007) 88:1547–60. 10.1016/j.apmr.2007.07.038 18047869

[37] Rintala DH Fiess RN Tan G Holmes SA Bruel BM. Effect of dronabinol on central neuropathic pain after spinal cord injury: a pilot study. Am J Phys Med Rehabil. (2010) 89:840–8. 10.1097/PHM.0b013e3181f1c4ec 20855984

[38] Lunn D Spiegelhalter D Thomas A Best N. The BUGS project: evolution, critique and future directions. Stat Med. (2009) 28:3049–67. 10.1002/sim.3680 19630097

[39] Shim SR Kim SJ. Intervention meta-analysis: application and practice using R software. Epidemiol Health. (2019) 41:e2019008. 10.4178/epih.e2019008 30999738 PMC6545497

[40] Naci H Ioannidis JP. Comparative effectiveness of exercise and drug interventions on mortality outcomes: metaepidemiological study. Br J Sports Med. (2015) 49:1414–22. 10.1136/bjsports-2015-f5577rep 26476429 PMC4680125

[41] Cheng J Cai M Shuai X Gao J Wang G Tao K. Multimodal treatments for resectable esophagogastric junction cancer: a systematic review and network meta-analysis. Ther Adv Med Oncol. (2019) 11:1–14. 10.1177/1758835919838963 31044021 PMC6446435

[42] Salanti G Ades AE Ioannidis JP. Graphical methods and numerical summaries for presenting results from multiple-treatment meta-analysis: an overview and tutorial. J Clin Epidemiol. (2011) 64:163–71. 10.1016/j.jclinepi.2010.03.016 20688472

[43] Hutton B Salanti G Caldwell DM Chaimani A Schmid CH Cameron C . The PRISMA extension statement for reporting of systematic reviews incorporating network meta-analyses of health care interventions: checklist and explanations. Ann Intern Med. (2015) 162:777–84. 10.7326/M14-2385 26030634

[44] Davis L Martin J. Studies upon spinal cord injuries; the nature and treatment of pain. J Neurosurg. (1947) 4:483–91. 10.3171/jns.1947.4.6.0483 20267936

[45] Alles SRA Smith PA. Etiology and pharmacology of neuropathic pain. Pharmacol Rev. (2018) 70:315–47. 10.1124/pr.117.014399 29500312

[46] Matak I Lacković Z. Botulinum toxin A, brain and pain. Prog Neurobiol. (2014) 119–20:39–59. 10.1016/j.pneurobio.2014.06.001 24915026

[47] Binder A Baron R. The pharmacological therapy of chronic neuropathic pain. Dtsch Arztebl Int. (2016) 113:616–25. 10.3238/arztebl.2016.0616 27697147 PMC5541246

[48] Favre-Guilmard C Auguet M Chabrier PE. Different antinociceptive effects of botulinum toxin type A in inflammatory and peripheral polyneuropathic rat models. Eur J Pharmacol. (2009) 617:48–53. 10.1016/j.ejphar.2009.06.047 19576881

[49] Park J Chung ME. Botulinum toxin for central neuropathic pain. Toxins. (2018) 10:224. 10.3390/toxins10060224 29857568 PMC6024683

[50] Finnerup NB Attal N Haroutounian S McNicol E Baron R Dworkin RH . Pharmacotherapy for neuropathic pain in adults: a systematic review and meta-analysis. Lancet Neurol. (2015) 14:162–73. 10.1016/S1474-4422(14)70251-0 25575710 PMC4493167

[51] Oh HM Chung ME. Botulinum toxin for neuropathic pain: a review of the literature. Toxins. (2015) 7:3127–54. 10.3390/toxins7083127 26287242 PMC4549742

[52] Yaksh TL Fisher CJ Hockman TM Wiese AJ. Current and future issues in the development of spinal agents for the management of pain. Curr Neuropharmacol. (2017) 15:232–59. 10.2174/1570159X14666160307145542 26861470 PMC5412694

[53] Wheeler A Smith HS. Botulinum toxins: mechanisms of action, antinociception and clinical applications. Toxicology. (2013) 306:124–46. 10.1016/j.tox.2013.02.006 23435179

[54] Herrero Babiloni A Kapos FP Nixdorf DR. Intraoral administration of botulinum toxin for trigeminal neuropathic pain. Oral Surg Oral Med Oral Pathol Oral Radiol. (2016) 121:e148–53. 10.1016/j.oooo.2016.03.013 27181448

[55] Ngeow WC Nair R. Injection of botulinum toxin type A (BOTOX) into trigger zone of trigeminal neuralgia as a means to control pain. Oral Surg Oral Med Oral Pathol Oral Radiol Endod. (2010) 109:e47–50. 10.1016/j.tripleo.2009.03.021 20219585

[56] Li G Lv CA Tian L Jin LJ Sun P Zhao W. A randomized controlled trial of botulinum toxin A for treating neuropathic pain in patients with spinal cord injury. Medicine. (2017) 96:e6919. 10.1097/MD.0000000000006919 28514309 PMC5440146

[57] Siongco PRL Rosales RL Moore AP Freynhagen R Arimura K Kanovsky P . Botulinum neurotoxin injections for muscle-based (dystonia and spasticity) and non-muscle-based (neuropathic pain) pain disorders: a meta-analytic study. J Neural Transm (Vienna). (2020) 127:935–51. 10.1007/s00702-020-02163-5 32146504

[58] Park J Park HJ. Botulinum toxin for the treatment of neuropathic pain. Toxins. (2017) 9:260. 10.3390/toxins9090260 28837075 PMC5618193

[59] Patel TP Ventre SC Geddes-Klein D Singh PK Meaney DF. Single-neuron NMDA receptor phenotype influences neuronal rewiring and reintegration following traumatic injury. J Neurosci. (2014) 34:4200–13. 10.1523/JNEUROSCI.4172-13.2014 24647941 PMC3960464

[60] Hershman DL Lacchetti C Dworkin RH Lavoie Smith EM Bleeker J Cavaletti G . Prevention and management of chemotherapy-induced peripheral neuropathy in survivors of adult cancers: American society of clinical oncology clinical practice guideline. J Clin Oncol. (2014) 32:1941–67. 10.1200/JCO.2013.54.0914 24733808

[61] Deng M Chen SR Pan HL. Presynaptic NMDA receptors control nociceptive transmission at the spinal cord level in neuropathic pain. Cell Mol Life Sci. (2019) 76:1889–99. 10.1007/s00018-019-03047-y 30788514 PMC6482077

[62] Luo C Kuner T Kuner R. Synaptic plasticity in pathological pain. Trends Neurosci. (2014) 37:343–55. 10.1016/j.tins.2014.04.002 24833289

[63] Zhuo M. Neural mechanisms underlying anxiety-chronic pain interactions. Trends Neurosci. (2016) 39:136–45. 10.1016/j.tins.2016.01.006 26878750

[64] Subramaniam K Subramaniam B Steinbrook RA. Ketamine as adjuvant analgesic to opioids: a quantitative and qualitative systematic review. Anesth Analg. (2004) 99:482–95. 10.1213/01.ANE.0000118109.12855.07 15271729

[65] Sator-Katzenschlager S Deusch E Maier P Spacek A Kress HG. The long-term antinociceptive effect of intrathecal S(+)-ketamine in a patient with established morphine tolerance. Anesth Analg. (2001) 93:1032–4. 10.1097/00000539-200110000-00047 11574378

[66] Backonja M Arndt G Gombar KA Check B Zimmermann M. Response of chronic neuropathic pain syndromes to ketamine: a preliminary study. Pain. (1994) 56:51–7. 10.1016/0304-3959(94)90149-X 8159441

[67] Kreutzwiser D Tawfic QA. Expanding role of NMDA receptor antagonists in the management of pain. CNS Drugs. (2019) 33:347–74. 10.1007/s40263-019-00618-2 30826987

[68] Chang CY Challa CK Shah J Eloy JD. Gabapentin in acute postoperative pain management. Biomed Res Int. (2014) 2014:631756. 10.1155/2014/631756 24829909 PMC4009126

[69] Moore C Liedtke WB. Osmomechanical-sensitive TRPV channels in mammals. In: Emir TLR, editor. Neurobiology of TRP Channels. Boca Raton (FL): CRC Press/Taylor & Francis© 2018 by Taylor & Francis Group, LLC (2017). 29356489

[70] Lanneau C Green A Hirst WD Wise A Brown JT Donnier E . Gabapentin is not a GABAB receptor agonist. Neuropharmacology. (2001) 41:965–75. 10.1016/S0028-3908(01)00140-X 11747901

[71] Li X DiFiglia M. The recycling endosome and its role in neurological disorders. Prog Neurobiol. (2012) 97:127–41. 10.1016/j.pneurobio.2011.10.002 22037413

[72] DeFrates S Cook AM. Pharmacologic treatment of neuropathic pain following spinal cord injury. Orthopedics. (2011) 34:203–7. 10.3928/01477447-20110124-19 21410102

[73] Guay DR. Pregabalin in neuropathic pain: a more “pharmaceutically elegant” gabapentin? Am J Geriatr Pharmacother. (2005) 3:274–87. 10.1016/j.amjopharm.2005.12.008 16503325

[74] Davari M Amani B Amani B Khanijahani A Akbarzadeh A Shabestan R. Pregabalin and gabapentin in neuropathic pain management after spinal cord injury: a systematic review and meta-analysis. Korean J Pain. (2020) 33:3–12. 10.3344/kjp.2020.33.1.3 31888312 PMC6944364

[75] Attal N Cruccu G Haanpää M Hansson P Jensen TS Nurmikko T . EFNS guidelines on pharmacological treatment of neuropathic pain. Eur J Neurol. (2006) 13:1153–69. 10.1111/j.1468-1331.2006.01511.x 17038030

[76] Kranzler HR Hernandez-Avila CA Gelernter J. Polymorphism of the 5-HT1B receptor gene (HTR1B): strong within-locus linkage disequilibrium without association to antisocial substance dependence. Neuropsychopharmacology. (2002) 26:115–22. 10.1016/S0893-133X(01)00283-4 11751038

[77] Casas J Gibert-Rahola J Chover AJ Micó JA. Test-dependent relationship of the antidepressant and analgesic effects of amitriptyline. Methods Find Exp Clin Pharmacol. (1995) 17:583–8. 8786671

[78] Widerström-Noga E. Neuropathic pain and spinal cord injury: phenotypes and pharmacological management. Drugs. (2017) 77:967–84. 10.1007/s40265-017-0747-8 28451808

[79] Wernicke JF Gahimer J Yalcin I Wulster-Radcliffe M Viktrup L. Safety and adverse event profile of duloxetine. Expert Opin Drug Saf. (2005) 4:987–93. 10.1517/14740338.4.6.987 16255658

[80] Ziegler D Pritchett YL Wang F Desaiah D Robinson MJ Hall JA . Impact of disease characteristics on the efficacy of duloxetine in diabetic peripheral neuropathic pain. Diabetes Care. (2007) 30:664–9. 10.2337/dc06-2009 17327338

[81] Mullan LA Mularczyk EJ Kung LH Forouhan M Wragg JM Goodacre R . Increased intracellular proteolysis reduces disease severity in an ER stress-associated dwarfism. J Clin Invest. (2017) 127:3861–5. 10.1172/JCI93094 28920921 PMC5617653

[82] Payne CE Brown AR Theile JW Loucif AJ Alexandrou AJ Fuller MD . A novel selective and orally bioavailable Nav 1.8 channel blocker, PF-01247324, attenuates nociception and sensory neuron excitability. Br J Pharmacol. (2015) 172:2654–70. 10.1111/bph.13092 25625641 PMC4409913

[83] Campbell JN Meyer RA. Mechanisms of neuropathic pain. Neuron. (2006) 52:77–92. 10.1016/j.neuron.2006.09.021 17015228 PMC1810425

[84] Lim MY Chandramouleeswaran S Zagar TM Budwit D Anders CK. Isolated cranial mononeuropathy: an unusual initial presentation and disease progression of metastatic carcinoma of the breast. J Clin Oncol. (2013) 31:e294–6. 10.1200/JCO.2012.47.3322 23669219 PMC4878085

[85] Wulff H Christophersen P Colussi P Chandy KG Yarov-Yarovoy V. Antibodies and venom peptides: new modalities for ion channels. Nat Rev Drug Discov. (2019) 18:339–57. 10.1038/s41573-019-0013-8 30728472 PMC6499689

[86] Finnerup NB Otto M McQuay HJ Jensen TS Sindrup SH. Algorithm for neuropathic pain treatment: an evidence based proposal. Pain. (2005) 118:289–305. 10.1016/j.pain.2005.08.013 16213659

[87] Beaulieu P Rice AS. The pharmacology of cannabinoid derivatives: are there applications to treatment of pain? Ann Fr Anesth Reanim. (2002) 21:493–508. 10.1016/S0750-7658(02)00663-9 12134594

[88] Romero-Sandoval EA Kolano AL Alvarado-Vázquez PA. Cannabis and cannabinoids for chronic pain. Curr Rheumatol Rep. (2017) 19:67. 10.1007/s11926-017-0693-1 28983880

[89] Habeck M. Bayesian approach to inverse statistical mechanics. Phys Rev E Stat Nonlin Soft Matter Phys. (2014) 89:052113. 10.1103/PhysRevE.89.052113 25353745

