# Supplementary material for: Comparative Efficacy and Safety of 11 Drugs as Therapies for Adults With Neuropathic Pain After Spinal Cord Injury: A Bayesian Network Analysis Based on 20 Randomized Controlled Trials
Source: Front Neurol. 2022 Mar 21;13:818522. doi: 10.3389/fneur.2022.818522 (PMC8977449; doi:10.3389/fneur.2022.818522)
Supplement: Supplementary file 1 [file Data_Sheet_1.pdf]

Supplementary Material

Comparative Efficacy and Safety of 11 Drugs as Therapies for Adults with Neuropathic Pain after Spinal Cord Injury: A Bayesian Network Analysis Based on 20 Randomized Controlled Trials

1 PRISMA Checklist

Section/topic	#	Checklist item	Reported on page #
**TITLE**			
Title	1	Identify the report as a systematic review, meta-analysis, or both.	1
**ABSTRACT**			
Structured summary	2	Provide a structured summary including, as applicable: background; objectives; data sources; study eligibility criteria, participants, and interventions; study appraisal and synthesis methods; results; limitations; conclusions and implications of key findings; systematic review registration number.	2
**INTRODUCTION**			
Rationale	3	Describe the rationale for the review in the context of what is already known.	2-3
Objectives	4	Provide an explicit statement of questions being addressed with reference to participants, interventions, comparisons, outcomes, and study design (PICOS).	3
**METHODS**			
Protocol and registration	5	Indicate if a review protocol exists, if and where it can be accessed (e.g., Web address), and, if available, provide registration information including registration number.	5-6

Eligibility criteria	6	Specify study characteristics (e.g., PICOS, length of follow-up) and report characteristics (e.g., years considered, language, publication status) used as criteria for eligibility, giving rationale.	4
Information sources	7	Describe all information sources (e.g., databases with dates of coverage, contact with study authors to identify additional studies) in the search and date last searched.	3
Search	8	Present full electronic search strategy for at least one database, including any limits used, such that it could be repeated.	3
Study selection	9	State the process for selecting studies (i.e., screening, eligibility, included in systematic review, and, if applicable, included in the meta-analysis).	4-5
Data collection process	10	Describe method of data extraction from reports (e.g., piloted forms, independently, in duplicate) and any processes for obtaining and confirming data from investigators.	4-5
Data items	11	List and define all variables for which data were sought (e.g., PICOS, funding sources) and any assumptions and simplifications made.	5
Risk of bias in individual studies	12	Describe methods used for assessing risk of bias of individual studies (including specification of whether this was done at the study or outcome level), and how this information is to be used in any data synthesis.	5
Summary measures	13	State the principal summary measures (e.g., risk ratio, difference in means).	5
Synthesis of results	14	Describe the methods of handling data and combining results of studies, if done, including measures of consistency (e.g., I2) for each meta-analysis.	5
Risk of bias across studies	15	Specify any assessment of risk of bias that may affect the cumulative evidence (e.g., publication bias, selective reporting within studies).	5

Additional analyses	16	Describe methods of additional analyses (e.g., sensitivity or subgroup analyses, meta-regression), if done, indicating which were pre-specified.	
**RESULTS**			
Study selection	17	Give numbers of studies screened, assessed for eligibility, and included in the review, with reasons for exclusions at each stage, ideally with a flow diagram.	10
Study characteristics	18	For each study, present characteristics for which data were extracted (e.g., study size, PICOS, follow-up period) and provide the citations.	10
Risk of bias within studies	19	Present data on risk of bias of each study and, if available, any outcome level assessment (see item 12).	10
Results of individual studies	20	For all outcomes considered (benefits or harms), present, for each study: (a) simple summary data for each intervention group (b) effect estimates and confidence intervals, ideally with a forest plot.	10-11
Synthesis of results	21	Present results of each meta-analysis done, including confidence intervals and measures of consistency.	10-11
Risk of bias across studies	22	Present results of any assessment of risk of bias across studies (see Item 15).	10-11
Additional analysis	23	Give results of additional analyses, if done (e.g., sensitivity or subgroup analyses, meta-regression [see Item 16]).	
**DISCUSSION**			
Summary of evidence	24	Summarize the main findings including the strength of evidence for each main outcome; consider their relevance to key groups (e.g., healthcare providers, users, and policy makers).	12-16

Limitations	25	Discuss limitations at study and outcome level (e.g., risk of bias), and at review-level (e.g., incomplete retrieval of identified research, reporting bias).	16-17
Conclusions	26	18	17
**FUNDING**			
Funding	27	Describe sources of funding for the systematic review and other support (e.g., supply of data); role of funders for the systematic review.	17

Supplementary 0. PRISMA Checklist

Search from PubMed	
**Search Details**	**Results**
((("Spinal Cord Injuries"[MeSH Terms] OR ((((((((((((((((((((((((((((((((((((("spinal cord trauma"[Title/Abstract] OR "cord trauma spinal"[Title/Abstract]) OR (((("cone-rod dystrophies"[MeSH Terms] OR ("cone rod"[All Fields] AND "dystrophies"[All Fields])) OR "cone rod dystrophies"[All Fields]) OR "Cord"[All Fields]) AND "traumas spinal"[Title/Abstract])) OR "spinal cord traumas"[Title/Abstract]) OR "trauma spinal cord"[Title/Abstract]) OR (((((((("Injuries"[MeSH Subheading] OR "Injuries"[All Fields]) OR "Trauma"[All Fields]) OR "wounds and injuries"[MeSH Terms]) OR ("wounds"[All Fields] AND "Injuries"[All Fields])) OR "wounds and injuries"[All Fields]) OR "trauma s"[All Fields]) OR "Traumas"[All Fields]) AND "spinal cord"[Title/Abstract])) OR "myelopathy traumatic"[Title/Abstract]) OR (((((((("spinal cord diseases"[MeSH Terms] OR (("Spinal"[All Fields] AND "Cord"[All Fields]) AND "diseases"[All Fields])) OR "spinal cord diseases"[All Fields]) OR "Myelopathies"[All Fields]) OR "Myelopathy"[All Fields]) OR "bone marrow diseases"[MeSH Terms]) OR (("bone"[All Fields] AND "marrow"[All Fields]) AND "diseases"[All Fields])) OR "bone marrow diseases"[All Fields]) AND "Traumatic"[Title/Abstract])) OR "traumatic myelopathies"[Title/Abstract]) OR "traumatic myelopathy"[Title/Abstract]) OR "injuries spinal cord"[Title/Abstract]) OR "cord injuries spinal"[Title/Abstract]) OR "cord injury spinal"[Title/Abstract]) OR "injury spinal cord"[Title/Abstract]) OR "spinal cord injury"[Title/Abstract]) OR "spinal cord transection"[Title/Abstract]) OR "cord transection spinal"[Title/Abstract]) OR (((("cone-rod dystrophies"[MeSH Terms] OR ("cone rod"[All Fields] AND "dystrophies"[All Fields])) OR "cone rod dystrophies"[All Fields]) OR "Cord"[All Fields]) AND "transections spinal"[Title/Abstract])) OR "spinal cord transections"[Title/Abstract]) OR "transection spinal cord"[Title/Abstract]) OR "transections spinal cord"[Title/Abstract]) OR "spinal cord laceration"[Title/Abstract]) OR (((("cone-rod dystrophies"[MeSH Terms] OR ("cone rod"[All Fields] AND "dystrophies"[All Fields])) OR "cone rod dystrophies"[All Fields]) OR "Cord"[All Fields]) AND "laceration spinal"[Title/Abstract])) OR (((("cone-rod dystrophies"[MeSH Terms] OR ("cone rod"[All Fields] AND "dystrophies"[All Fields])) OR "cone rod dystrophies"[All Fields]) OR "Cord"[All Fields]) AND "lacerations spinal"[Title/Abstract])) OR "laceration spinal cord"[Title/Abstract]) OR (((((("Lacerations"[MeSH Terms] OR "Lacerations"[All Fields]) OR "lacerate"[All Fields]) OR "lacerated"[All Fields]) OR "lacerating"[All Fields]) OR "Laceration"[All Fields]) AND "spinal cord"[Title/Abstract])) OR ((("spinal cord"[MeSH Terms] OR ("Spinal"[All Fields] AND "Cord"[All Fields])) OR "spinal cord"[All Fields]) AND "Lacerations"[Title/Abstract])) OR "post traumatic myelopathy"[Title/Abstract]) OR (((((((("spinal cord diseases"[MeSH Terms] OR (("Spinal"[All Fields] AND "Cord"[All Fields]) AND "diseases"[All Fields])) OR "spinal cord diseases"[All Fields]) OR "Myelopathies"[All Fields]) OR "Myelopathy"[All Fields]) OR "bone marrow diseases"[MeSH Terms]) OR (("bone"[All Fields] AND "marrow"[All Fields]) AND "diseases"[All Fields])) OR "bone marrow diseases"[All Fields]) AND "Post-Traumatic"[Title/Abstract])) OR (((((((("spinal cord diseases"[MeSH Terms] OR (("Spinal"[All Fields] AND "Cord"[All Fields]) AND "diseases"[All Fields])) OR "spinal cord diseases"[All Fields]) OR "Myelopathies"[All Fields]) OR "Myelopathy"[All Fields]) OR "bone marrow diseases"[MeSH Terms]) OR (("bone"[All Fields] AND "marrow"[All Fields]) AND "diseases"[All Fields])) OR "bone marrow diseases"[All Fields]) AND "Post-Traumatic"[Title/Abstract])) OR "post traumatic myelopathy"[Title/Abstract]) OR "post traumatic myelopathies"[Title/Abstract]) OR "spinal cord contusion"[Title/Abstract]) OR "contusion spinal cord"[Title/Abstract]) OR "contusions spinal cord"[Title/Abstract]) OR (((("cone-rod dystrophies"[MeSH Terms] OR ("cone rod"[All Fields] AND "dystrophies"[All Fields])) OR "cone rod dystrophies"[All Fields]) OR "Cord"[All Fields]) AND "contusion spinal"[Title/Abstract])) OR "cord contusions spinal"[Title/Abstract]) OR "spinal cord contusions"[Title/Abstract])) AND ("Neuralgia"[MeSH Terms] OR ((("Neuralgias"[Title/Abstract] OR "neuropathic pain"[Title/Abstract]) OR "neuropathic pains"[Title/Abstract]) OR "pain neuropathic"[Title/Abstract]) OR "pains neuropathic"[Title/Abstract]) OR "Neurodynia"[Title/Abstract]) OR "neuralgia atypical"[Title/Abstract]) OR "atypical neuralgia"[Title/Abstract]) OR "atypical neuralgias"[Title/Abstract]) OR "neuralgias atypical"[Title/Abstract]) OR ((("Neuralgia"[MeSH Terms] OR "Neuralgia"[All Fields]) OR "Neuralgias"[All Fields]) AND "iliohypogastric nerve"[Title/Abstract])) OR ("Iliohypogastric"[All Fields] AND "nerve neuralgia"[Title/Abstract])) OR ("Iliohypogastric"[All Fields] AND "nerve neuralgias"[Title/Abstract])) OR ((((("Nerve"[All Fields] OR "nerve s"[All Fields]) OR "nerved"[All Fields]) OR "nerves"[All Fields]) AND (("Neuralgia"[MeSH Terms] OR "Neuralgia"[All Fields]) OR "Neuralgias"[All Fields])) AND "Iliohypogastric"[Title/Abstract])) OR ((((("Nerve"[All Fields] OR "nerve s"[All Fields]) OR "nerved"[All Fields]) OR "nerves"[All Fields]) AND (("Neuralgia"[MeSH Terms] OR "Neuralgia"[All Fields]) OR "Neuralgias"[All Fields])) AND "Iliohypogastric"[Title/Abstract])) OR ((("Neuralgia"[MeSH Terms] OR "Neuralgia"[All Fields]) OR "Neuralgias"[All Fields]) AND "iliohypogastric nerve"[Title/Abstract])) OR "paroxysmal nerve pain"[Title/Abstract]) OR (((("Nerve"[All Fields] OR "nerve s"[All Fields]) OR "nerved"[All Fields]) OR "nerves"[All Fields]) AND "pain paroxysmal"[Title/Abstract])) OR (((("Nerve"[All Fields] OR "nerve s"[All Fields]) OR "nerved"[All Fields]) OR "nerves"[All Fields]) AND "pains paroxysmal"[Title/Abstract])) OR (("Pain"[MeSH Terms] OR "Pain"[All Fields]) AND "paroxysmal nerve"[Title/Abstract])) OR (((((("Pain"[MeSH Terms] OR "Pain"[All Fields]) OR "painful"[All Fields]) OR "Pains"[All Fields]) OR "pain s"[All Fields]) OR "painfulness"[All Fields]) AND "paroxysmal nerve"[Title/Abstract])) OR (((("paroxysm"[All Fields] OR "Paroxysmal"[All Fields]) OR "paroxysmally"[All Fields]) OR "paroxysms"[All Fields]) AND "nerve pains"[Title/Abstract])) OR ((("Neuralgia"[MeSH Terms] OR "Neuralgia"[All Fields]) OR "Neuralgias"[All Fields]) AND "Perineal"[Title/Abstract])) OR ((("Neuralgia"[MeSH Terms] OR "Neuralgia"[All Fields]) OR "Neuralgias"[All Fields]) AND "Perineal"[Title/Abstract])) OR "perineal neuralgia"[Title/Abstract]) OR "perineal neuralgias"[Title/Abstract]) OR ((("Neuralgia"[MeSH Terms] OR "Neuralgia"[All Fields]) OR "Neuralgias"[All Fields]) AND "Stump"[Title/Abstract])) OR ((("Neuralgia"[MeSH Terms] OR "Neuralgia"[All Fields]) OR "Neuralgias"[All Fields]) AND "Stump"[Title/Abstract])) OR (((((("amputation stumps"[MeSH Terms] OR ("amputation"[All Fields] AND "stumps"[All Fields])) OR "amputation stumps"[All Fields]) OR "Stump"[All Fields]) OR "stumps"[All Fields]) OR "stump s"[All Fields]) AND "Neuralgia"[Title/Abstract])) OR (((((("amputation stumps"[MeSH Terms] OR ("amputation"[All Fields] AND "stumps"[All Fields])) OR "amputation stumps"[All Fields]) OR "Stump"[All Fields]) OR "stumps"[All Fields]) OR "stump s"[All Fields]) AND "Neuralgias"[Title/Abstract])) OR "neuralgia supraorbital"[Title/Abstract]) OR ((("Neuralgia"[MeSH Terms] OR "Neuralgia"[All Fields]) OR "Neuralgias"[All Fields]) AND "Supraorbital"[Title/Abstract])) OR "supraorbital neuralgia"[Title/Abstract]) OR (("Supraorbital"[All Fields] OR "supraorbitals"[All Fields]) AND "Neuralgias"[Title/Abstract])) OR "neuralgia vidian"[Title/Abstract]) OR ((("Neuralgia"[MeSH Terms] OR "Neuralgia"[All Fields]) OR "Neuralgias"[All Fields]) AND "Vidian"[Title/Abstract])) OR ("Vidian"[All Fields] AND "Neuralgia"[Title/Abstract])) OR ("Vidian"[All Fields] AND "Neuralgias"[Title/Abstract])) OR "nerve pain"[Title/Abstract]) OR "nerve pains"[Title/Abstract]) OR "pain nerve"[Title/Abstract]) OR (((((("Pain"[MeSH Terms] OR "Pain"[All Fields]) OR "painful"[All Fields]) OR "Pains"[All Fields]) OR "pain s"[All Fields]) OR "painfulness"[All Fields]) AND "Nerve"[Title/Abstract])) OR ((("Neuralgia"[MeSH Terms]	161

OR "Neuralgia"[All Fields]) OR "Neuralgias"[All Fields]) AND "Ilioinguinal"[Title/Abstract])) OR "ilioinguinal neuralgia"[Title/Abstract]) OR "ilioinguinal neuralgias"[Title/Abstract]) OR ((("Neuralgia"[MeSH Terms] OR "Neuralgia"[All Fields]) OR "Neuralgias"[All Fields]) AND "Ilioinguinal"[Title/Abstract])))) AND ("Therapeutics"[MeSH Terms] OR (((("Therapeutic"[Title/Abstract] OR "Therapy"[Title/Abstract]) OR "Therapies"[Title/Abstract]) OR "Treatment"[Title/Abstract]) OR "Treatments"[Title/Abstract]))) AND (("randomized controlled trial"[Publication Type] OR "randomized"[Title/Abstract]) OR "placebo"[Title/Abstract])	

Supplementary 1. The Search Strategies Used in PubMed

Search from Embase

Embase®

Embase Session Results

No.	Query	Results
#11	#3 AND #6 AND #8 AND #10	232
#10	'randomized controlled trial':ab,ti OR 'randomized':ab,ti OR 'placebo':ab,ti OR 'double-blind':ab,ti	932,651
#9	#7 OR #8	12,981,704
#8	'therapeutic':ab,ti OR 'therapy':ab,ti OR 'therapies':ab,ti OR 'treatment':ab,ti OR 'treatments':ab,ti	8,267,155
#7	'therapy'/exp	8,767,244
#6	#4 OR #5	116,528
#5	'neuralgias':ab,ti OR 'neuropathic pain':ab,ti OR 'neuropathic pains':ab,ti OR 'pain, neuropathic':ab,ti OR 'pains, neuropathic':ab,ti OR 'neurodynia':ab,ti OR 'neurodynias':ab,ti OR 'neuralgia, atypical':ab,ti OR 'atypical neuralgia':ab,ti OR 'atypical neuralgias':ab,ti OR 'neuralgias, atypical':ab,ti OR 'neuralgia, iliohypogastric nerve':ab,ti OR 'iliohypogastric nerve neuralgia':ab,ti OR 'iliohypogastric nerve neuralgias':ab,ti OR 'nerve neuralgia, iliohypogastric':ab,ti OR 'nerve neuralgias, iliohypogastric':ab,ti OR 'neuralgias, iliohypogastric nerve':ab,ti OR 'paroxysmal nerve pain':ab,ti OR 'nerve pain, paroxysmal':ab,ti OR 'nerve pains, paroxysmal':ab,ti OR 'pain, paroxysmal nerve':ab,ti OR 'pains, paroxysmal nerve':ab,ti OR 'paroxysmal nerve pains':ab,ti OR 'neuralgia, perineal':ab,ti OR 'neuralgias, perineal':ab,ti OR 'perineal neuralgia':ab,ti OR 'perineal neuralgias':ab,ti OR 'neuralgia, stump':ab,ti OR 'neuralgias, stump':ab,ti OR 'stump neuralgia':ab,ti OR 'stump neuralgias':ab,ti OR 'neuralgia, supraorbital':ab,ti OR 'neuralgias, supraorbital':ab,ti OR 'supraorbital neuralgia':ab,ti OR 'supraorbital neuralgias':ab,ti OR 'neuralgia, vidian':ab,ti OR 'neuralgias, vidian':ab,ti OR 'vidian neuralgia':ab,ti OR 'vidian neuralgias':ab,ti OR 'nerve pain':ab,ti OR 'nerve pains':ab,ti OR 'pain, nerve':ab,ti OR 'pains, nerve':ab,ti OR 'neuralgia, ilioinguinal':ab,ti OR 'ilioinguinal neuralgia':ab,ti OR 'ilioinguinal neuralgias':ab,ti OR 'neuralgias, ilioinguinal':ab,ti	30,546
#4	'neuralgia'/exp	110,109
#3	#1 OR #2	84,083
#2	'spinal cord trauma':ab,ti OR 'cord trauma, spinal':ab,ti OR 'cord traumas, spinal':ab,ti OR 'spinal cord traumas':ab,ti OR 'trauma, spinal cord':ab,ti OR 'traumas, spinal cord':ab,ti OR 'myelopathy, traumatic':ab,ti OR 'myelopathies, traumatic':ab,ti OR 'traumatic myelopathies':ab,ti OR 'traumatic myelopathy':ab,ti OR 'injuries, spinal cord':ab,ti OR 'cord injuries, spinal':ab,ti OR 'cord injury, spinal':ab,ti OR 'injury, spinal cord':ab,ti OR 'spinal cord injury':ab,ti OR 'spinal cord transection':ab,ti OR 'cord transection, spinal':ab,ti OR 'cord transections, spinal':ab,ti OR 'spinal cord transections':ab,ti OR 'transection, spinal cord':ab,ti OR 'transections, spinal cord':ab,ti OR 'spinal cord laceration':ab,ti OR 'cord laceration, spinal':ab,ti OR 'cord lacerations, spinal':ab,ti OR 'laceration, spinal cord':ab,ti OR 'lacerations, spinal cord':ab,ti OR 'spinal cord lacerations':ab,ti OR 'post-traumatic myelopathy':ab,ti OR 'myelopathies, post-traumatic':ab,ti OR 'myelopathy, post-traumatic':ab,ti OR 'post traumatic myelopathy':ab,ti OR 'post-traumatic myelopathies':ab,ti OR 'spinal cord contusion':ab,ti OR 'contusion, spinal cord':ab,ti OR 'contusions, spinal cord':ab,ti OR 'cord contusion, spinal':ab,ti OR 'cord contusions, spinal':ab,ti OR 'spinal cord contusions':ab,ti	46,287
#1	'spinal cord injury'/exp	78,598

ELSEVIER

© 2020 RELX Intellectual Properties SA. All rights reserved.
Embase, RELX Group and the RE symbol are trademarks of RELX Intellectual Properties SA, used under license.

Supplementary 2. The Search Strategies Used in Embase.

Supplementary Table 1. Primary Outcome - Pain Relief for Almost 4 Weeks.

**Primary outcomes-efficacy (pain relief) [almost 4 weeks)]**	**ID**	**Treatment**	**n**	**Mean change**	**sd**
Nct2012	2	cannabinoids	55	-0.74	1.12
	1	placebo	59	-0.69	1.39
Cardenas2013	3	pregabalin	107	-1.64	1.61
	1	placebo	103	-1.03	1.53
Agarwal2017	4	amitriptyline	74	-0.43	0.18
	5	lamotrigine	73	-0.44	0.21
Amr2010	6	ketamine	20	-42.2	4.4
	7	gabapentin	20	-43.5	2.3
Amr2011	6	ketamine	20	-48.3	9.23
	7	gabapentin	20	-32.15	9.95
Andresen2016	2	cannabinoids	34	-0.4	1.51
	1	placebo	34	-0.7	1.57
Salinas2012	8	carbamazepine	23	-14.7	31.59
	1	placebo	21	-14.5	31.9
Siddall2006	3	pregabalin	69	-1.92	1.84
	1	placebo	67	-0.46	1.85
Tai2016	7	gabapentin	7	-0.4	3.12
	1	placebo	7	2.3	4.4
Vranken2008	3	pregabalin	17	-2.5	2.59

	1	placebo	16	-0.1	1.73
Vranken2011	9	duloxetine	18	-2.1	1.74
	1	placebo	18	-1.1	1.47
Yilmaz2015	3	pregabalin	15	-3.22	2.95
	7	gabapentin	15	-2.25	2.41
chun2019	10	BTXA	5	-18.6	16.8
	1	placebo	3	-2.6	14.6
Finnerup2009	11	levetiracetam	34	0	4.36
	1	placebo	32	1	4.12
Han2016	10	BTXA	20	-18.6	18.22
	1	placebo	20	3.4	15.1
Kaydok2014	7	gabapentin	21	-4.21	1.24
	3	pregabalin	19	-4.69	1.19
Levendoglu2004	7	gabapentin	20	-3.7	1.01
	1	placebo	20	-0.7	3.72
Norrbrink2009	12	tramadol	23	-1	2.32
	1	placebo	12	-0.25	1.58
Rintala2007	4	amitriptyline	28	-2.14	2.15
	7	gabapentin	26	-0.75	2.59
	1	placebo	25	-0.49	2.39
Rintala2010	2	cannabinoids	7	-0.2	0.67

1	placebo	5		-0.14	1.25

Supplementary Table 2. Primary Outcome - Any Adverse Events.

**Primary outcomes - safety (any adverse events)**	**ID**	**Treatment**	**n**	**r**
Nct2012	2	cannabinoids	56	46
	1	placebo	60	29
Cardenas2013	3	pregabalin	112	78
	1	placebo	107	56
Agarwal2017	4	amitriptyline	74	10
	5	lamotrigine	73	0
Amr2010	6	ketamine	20	8
	7	gabapentin	20	2
Amr2011	6	ketamine	20	0
	7	gabapentin	20	6
Andresen2016	2	cannabinoids	34	7
	1	placebo	34	2
Salinas2012	8	carbamazepine	23	11
	1	placebo	21	5
Siddall2006	3	pregabalin	70	67
	1	placebo	67	50
Tai2016	7	gabapentin	7	1

	1	placebo	7	0
Vranken2008	3	pregabalin	17	2
	1	placebo	16	2
Vranken2011	9	duloxetine	18	12
	1	placebo	18	2
Yilmaz2015	3	pregabalin	15	2
	7	gabapentin	15	0
chun2019	10	BTX-A	6	1
	1	placebo	6	0
Finnerup2009	11	Levetiracetam	34	14
	1	placebo	32	11
Han2016	10	BTX-A	20	8
	1	placebo	20	9
Kaydok2014	7	gabapentin	24	16
	3	pregabalin	25	21
Levendoglu2004	7	gabapentin	20	13
	1	placebo	20	5
Norrbrink2009	12	tramadol	23	21
	1	placebo	12	7
Rintala2007	4	amitriptyline	28	10
	7	gabapentin	26	9

	1	placebo	25	8
Rintala2010	2	cannabinoids	7	5
	1	placebo	5	5

Supplementary Table 3. Secondary Outcome - Pain Relief for More Than 8 Weeks.

Primary outcomes-efficacy (pain relief) [more than 8 weeks)	ID	Treatment	n	Mean change	sd
Cardenas2013	2	pregabalin	89	-2.17	1.78
	1	placebo	90	-1.36	1.87
Amr2011	3	ketamine	20	-32.2	8.94
	4	gabapentin	20	-28.95	10.14
Andresen2016	5	cannabinoids	34	-0.4	1.51
	1	Placebo	34	-0.7	1.57
Salinas2012	6	carbamazepine	23	-8.4	33.45
	1	placebo	20	-5	29.67
Siddall2006	2	pregabalin	69	-1.92	1.84
	1	placebo	67	-0.46	1.85
Vranken2011	7	duloxetine	18	-2.1	1.74
	1	placebo	18	-1.1	1.47
Yilmaz2015	2	pregabalin	10	-4.89	1.84
	4	gabapentin	11	-3.6	1.38

chun2019	8	BTX-A	6	-1.67	2.59
	1	placebo	6	-1	0.95
Han2016	8	BTX-A	20	-21.3	23.82
	1	placebo	20	5.7	18.07
Levendoglu2004	4	gabapentin	20	-5.3	1.08
	1	placebo	20	-0.5	3.76
Rintala2007	9	amitriptyline	28	-2.14	2.15
	4	gabapentin	26	-0.75	2.59
	1	placebo	25	-0.49	2.39

Supplementary Table 4. Secondary Outcome - Mental or Sleep-Related Symptom Relief.

**Secondary outcomes - efficacy (mental or sleep-related symptom relief)**	**ID**	**Treatment**	**n**	**mean change**	**sd**
Nct2012	2	cannabinoids	55	-0.41	0.59
	1	placebo	59	-0.38	0.73
Cardenas2013	3	pregabalin	105	-10.8	16.7
	1	placebo	106	-5.8	16.21
Salinas2012	4	carbamazepine	23	-19.7	39.12
	1	placebo	21	-27.6	44.64
Siddall2006	3	pregabalin	69	-8.8	19.09
	1	placebo	67	-5.4	20.29
Vranken2008	3	pregabalin	17	-4.2	14.13

	1	placebo	16	1.6	14.75
Vranken2011	5	duloxetine	18	-5	11.73
	1	placebo	18	-2	13.83
Yilmaz2015	3	pregabalin	15	-2.81	16.06
	6	gabapentin	15	-8.36	21.57
Chun2019	7	BTX-A	6	-1.34	3.05
	1	placebo	6	0.67	3.19
Finnerup2009	8	levetiracetam	25	-1	6.33
	1	placebo	29	-0.5	6.33
Han2016	7	BTX-A	20	-0.9	3.68
	1	placebo	20	1	3.2
Kaydok2014	6	gabapentin	21	-3.79	1.72
	3	pregabalin	19	-3.84	1.44
Levendoglu2004	6	gabapentin	20	-0.56	0.17
	1	placebo	20	-0.13	0.5
Norrbrink2009	9	tramadol	23	-1	5.19
	1	placebo	12	0	5.11

Supplementary Table 5. Secondary Outcome - Serious Adverse Events.

**Secondary outcomes - safety [serious adverse events (SAE)]**	**ID**	**Treatment**	**n**	**r**
Nct2012	2	cannabinoids	56	3

	1	placebo	60	2
Cardenas2013	3	pregabalin	112	9
	1	placebo	107	10
Agarwal2017	4	amitriptyline	74	0
	5	lamotrigine	73	0
Amr2010	6	ketamine	20	0
	7	gabapentin	20	0
Amr2011	6	ketamine	20	0
	7	gabapentin	20	0
Andresen2016	2	cannabinoids	34	4
	1	placebo	34	2
Salinas2012	8	carbamazepine	23	0
	1	placebo	21	0
Siddall2006	3	pregabalin	70	2
	1	placebo	67	0
Vranken2008	3	pregabalin	17	0
	1	placebo	16	0
Vranken2011	9	duloxetine	18	0
	1	placebo	18	0
Yilmaz2015	3	pregabalin	15	0
	7	gabapentin	15	0

chun2019	10	BTX-A	6	0
	1	placebo	6	0
Finnerup2009	11	levetiracetam	34	9
	1	placebo	32	4
Han2016	10	BTX-A	20	0
	1	placebo	20	0
Kaydok2014	7	gabapentin	24	2
	3	pregabalin	25	5
Levendoglu2004	7	gabapentin	20	0
	1	placebo	20	0
Norrbrink2009	12	tramadol	23	16
	1	placebo	12	2
Rintala2007	4	amitriptyline	28	4
	7	gabapentin	26	5
	1	placebo	25	2
Rintala2010	2	cannabinoids	7	1
	1	placebo	5	1
Tai2016	7	gabapentin	7	0
	1	placebo	7	0

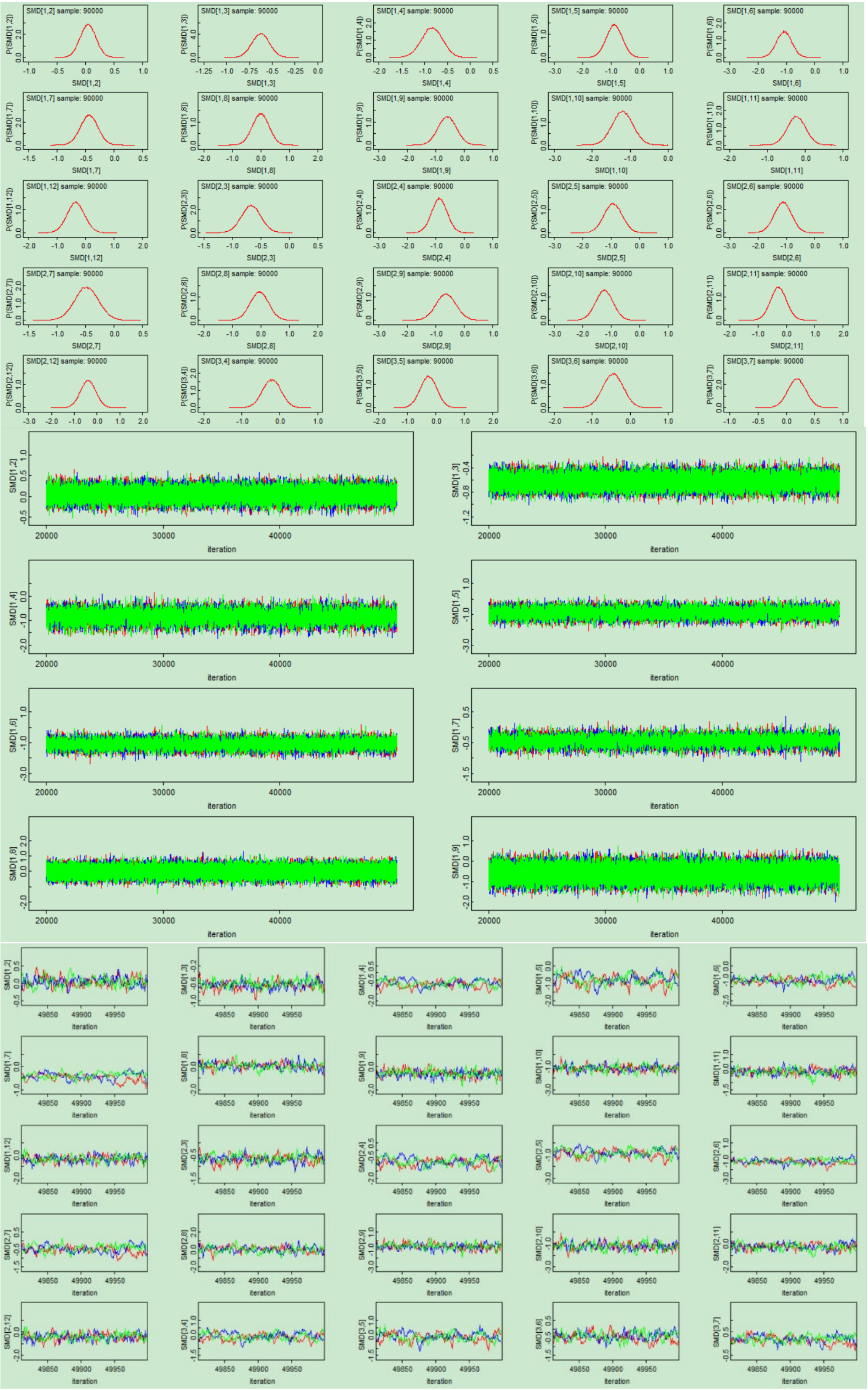

Supplementary FIGURE 1. The data Fitting Effect of Outcomes with Density, Trace, and History visualized via OpenBUGS.

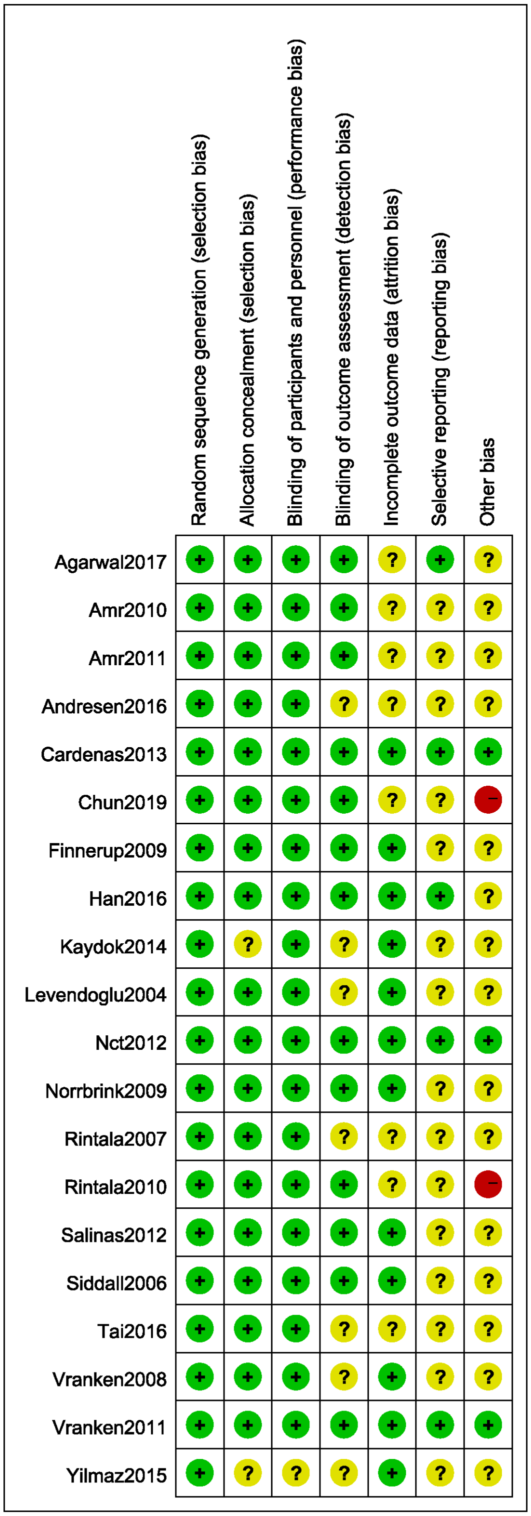

Supplementary FIGURE 2. Risk of Bias Summary

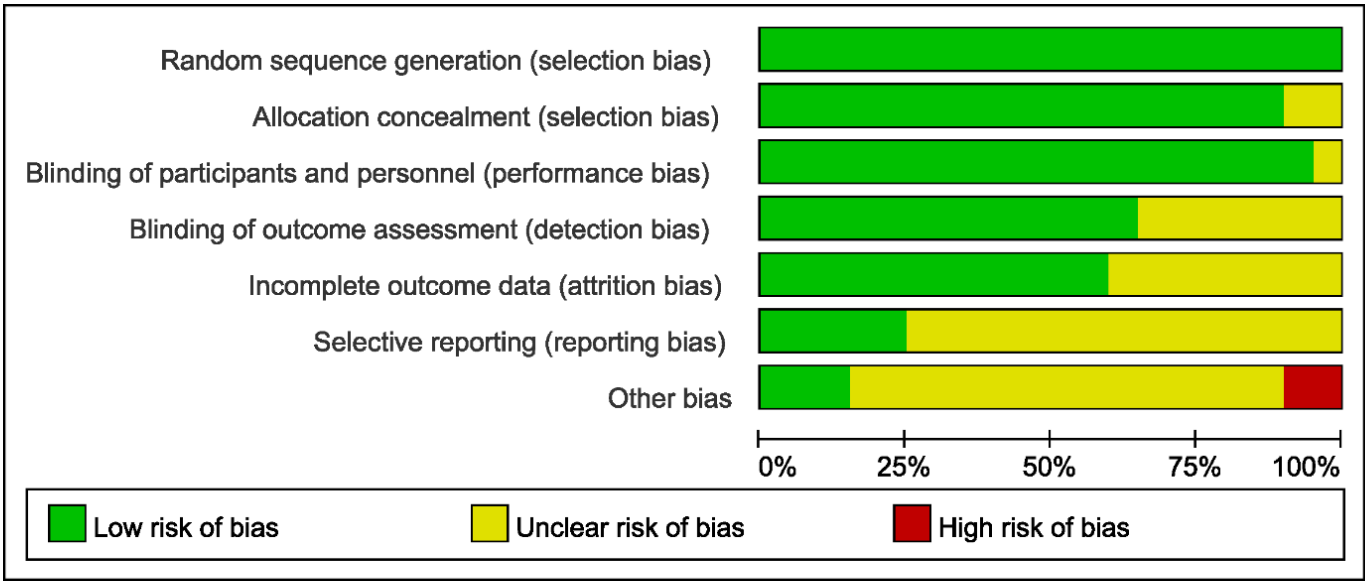

Supplementary FIGURE 3. Risk of Bias Graph.

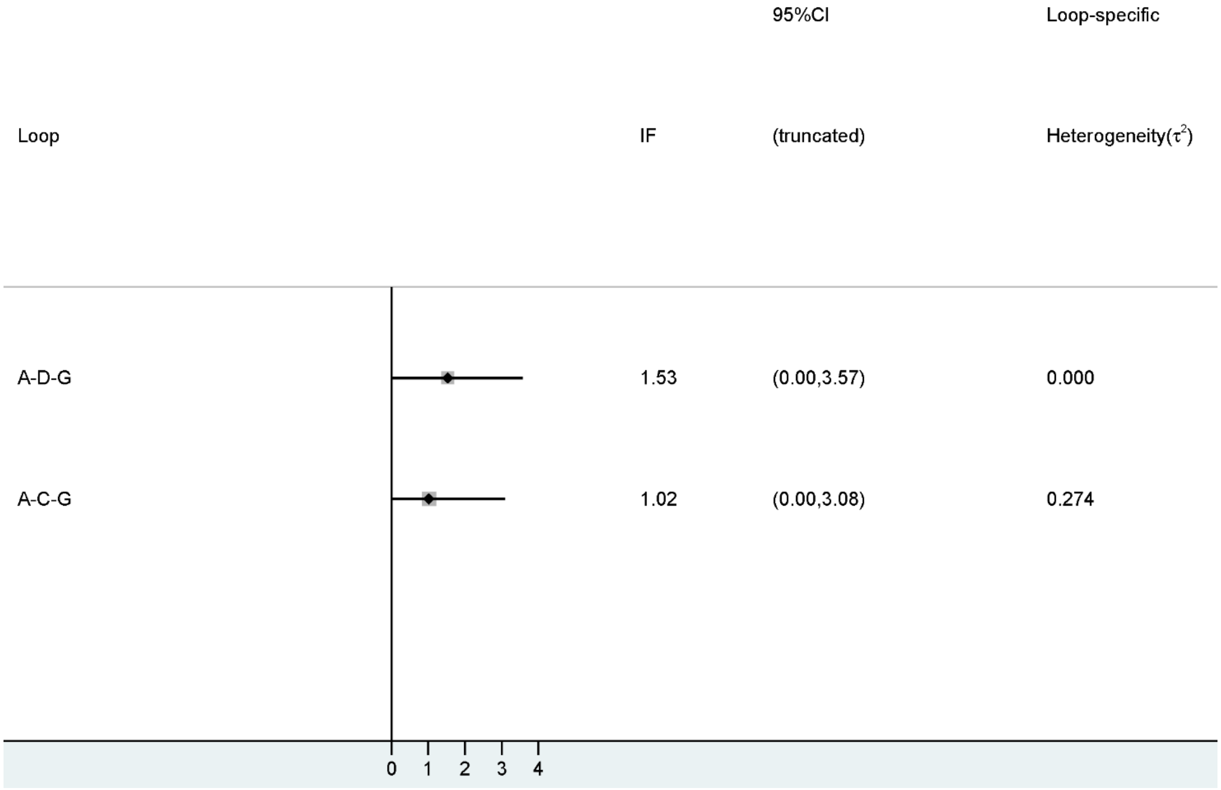

Supplementary FIGURE 4. The Node Splitting Method for Testing the Inconsistency of Primary Outcome (safety).

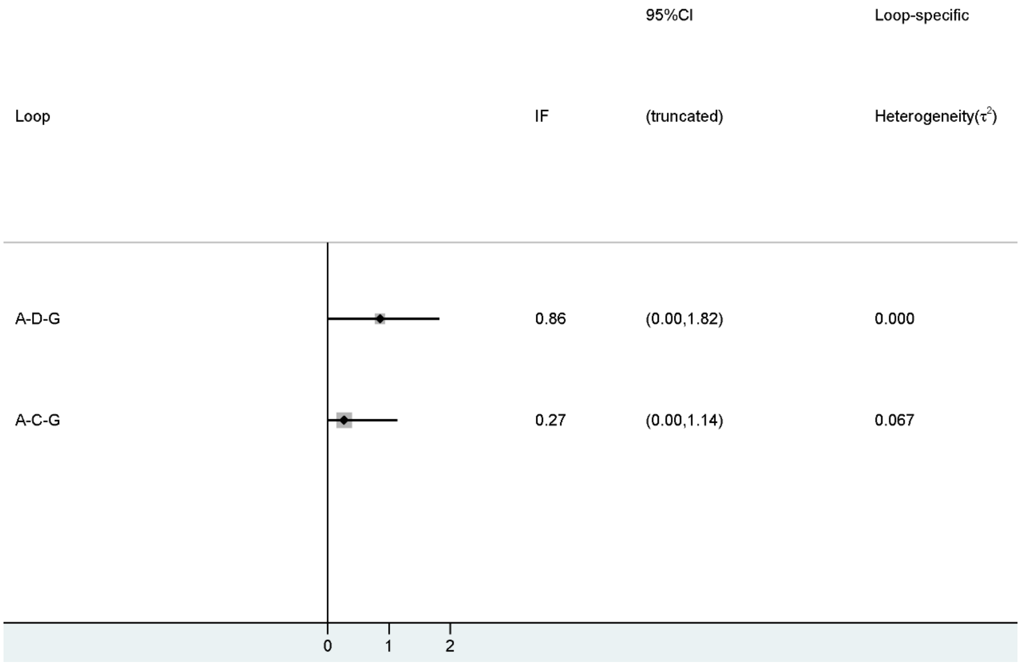

Supplementary FIGURE 5. The Node Splitting Method for Testing the Inconsistency of Primary Outcome (efficacy).

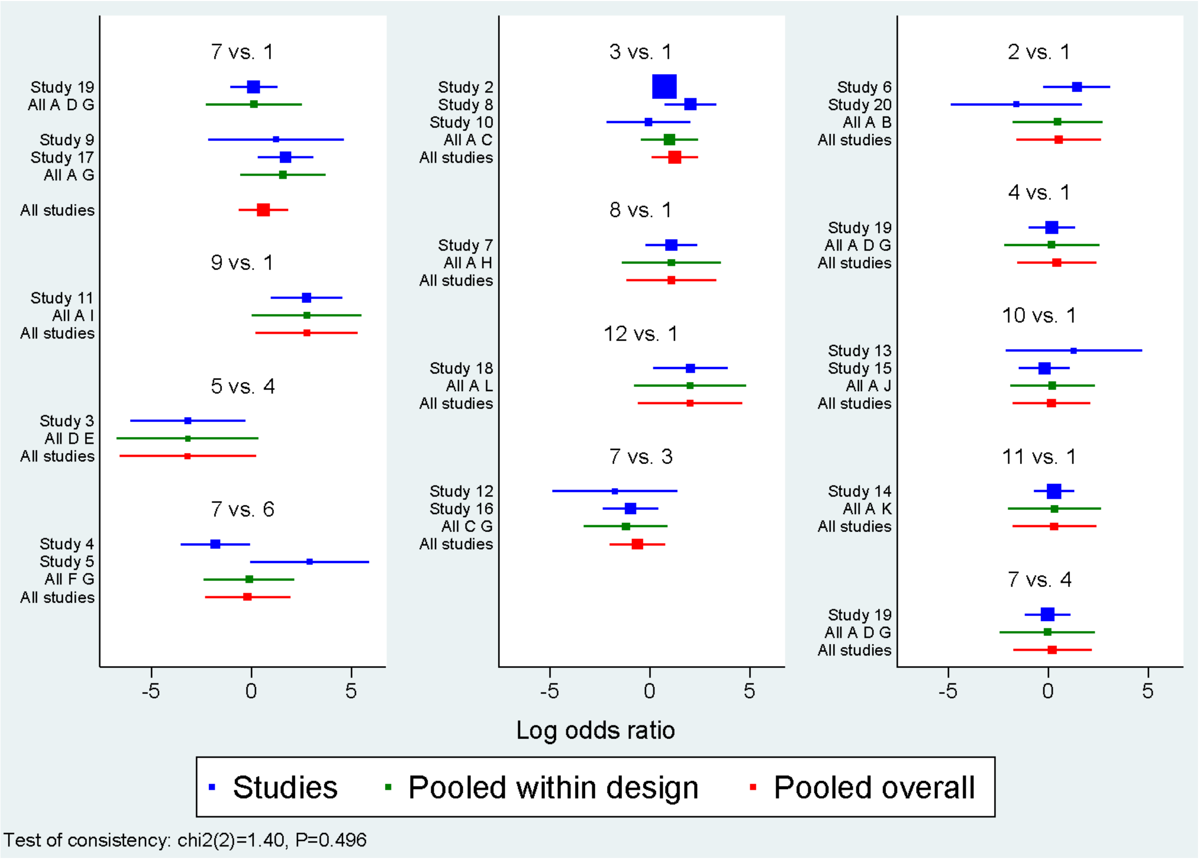

Supplementary FIGURE 6. Safety Pairwise and Network Meta-analysis of 11 Drugs and Placebo.

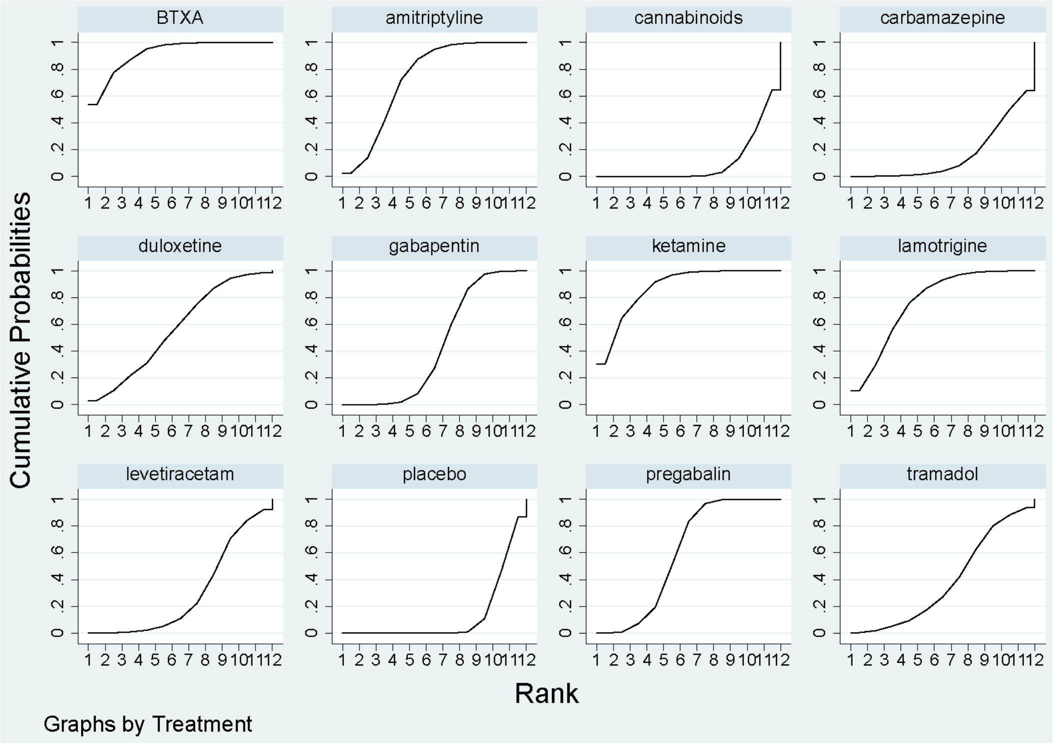

Supplementary FIGURE 7. The SUCRA of Primary Outcome (pain relief for around 4 weeks)

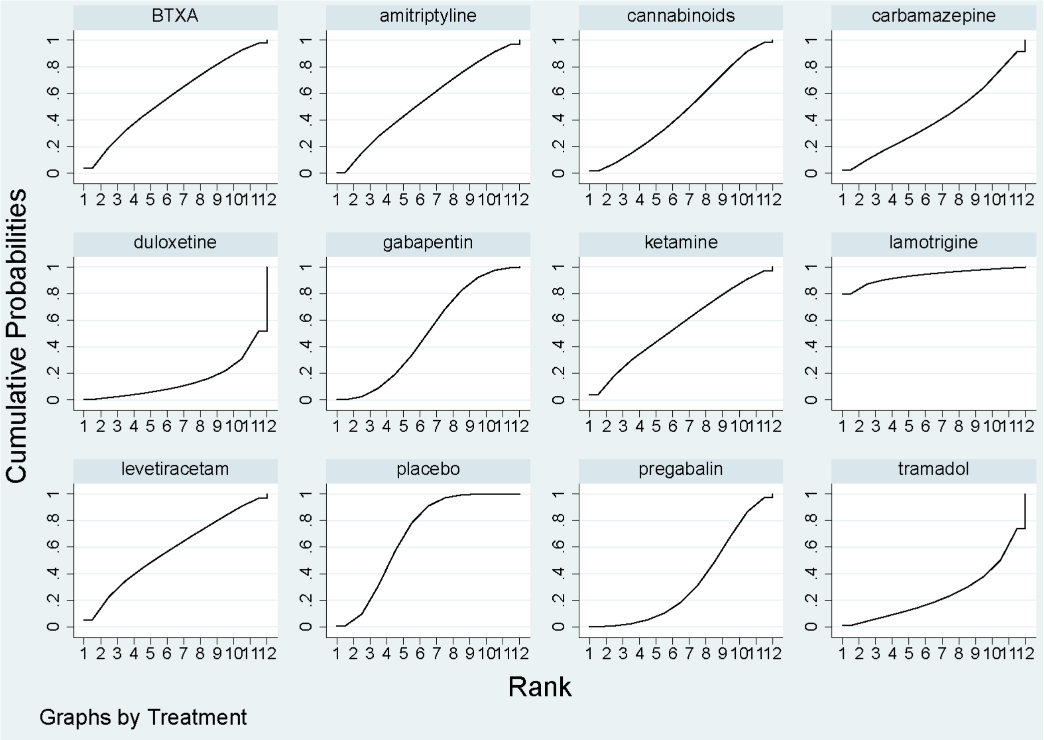

Supplementary FIGURE 8. The SUCRA of Primary Outcome (any adverse events).

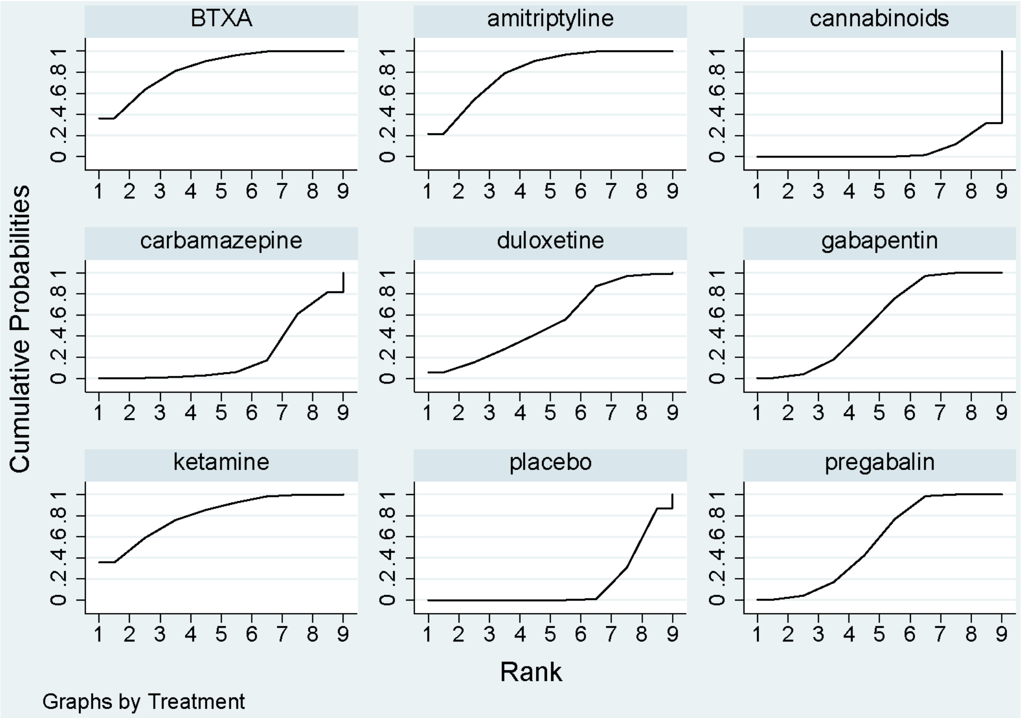

Supplementary FIGURE 9. The SUCRA of Secondary Outcome (pain relief for more than 8 weeks).

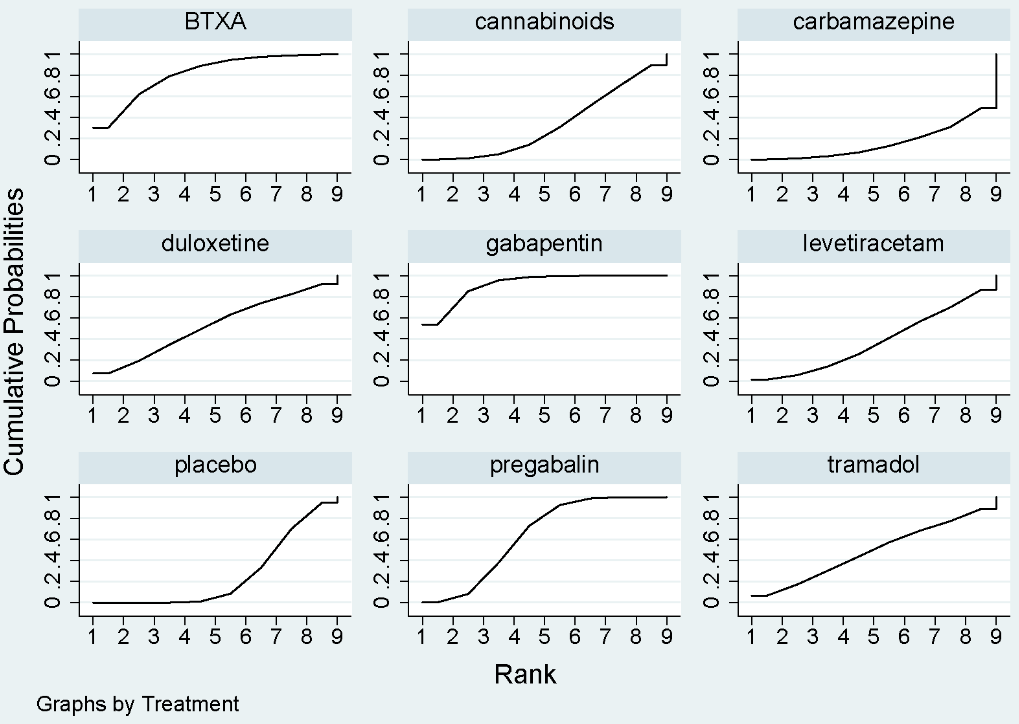

Supplementary FIGURE 10. The SUCRA of Secondary Outcome (mental or sleep-related symptom relief).

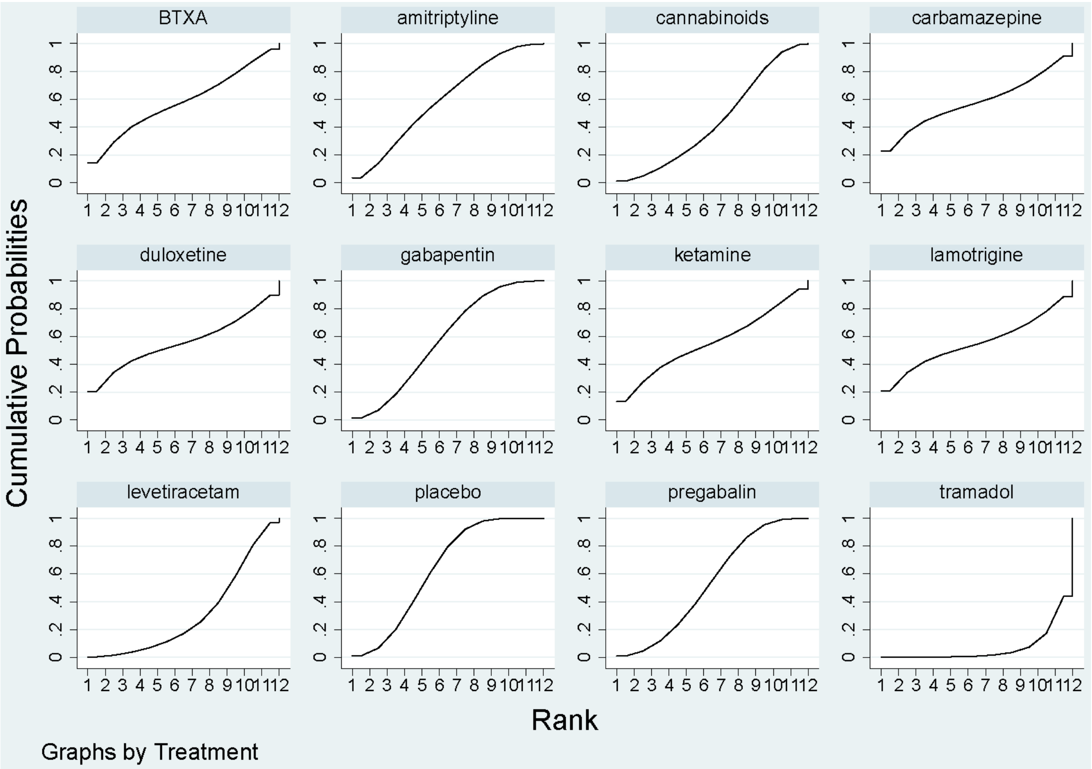

Supplementary FIGURE 21. The SUCRA of Secondary Outcome (serious adverse events).

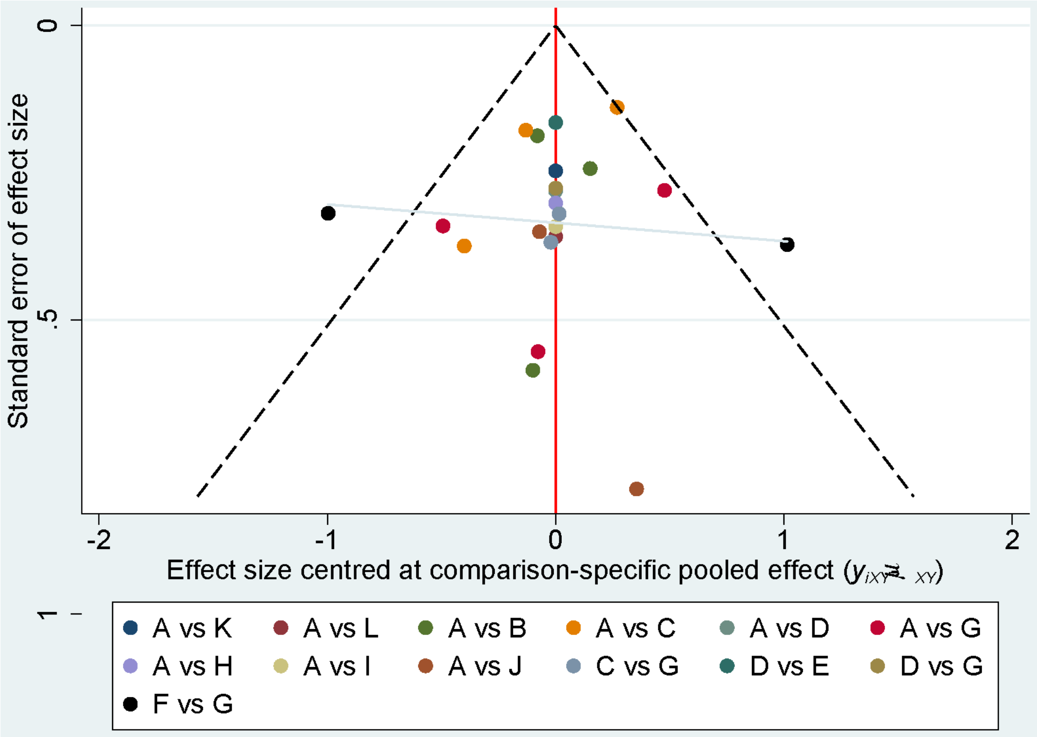

Supplementary FIGURE 32. The net-funnel of Primary Outcome-Efficacy.

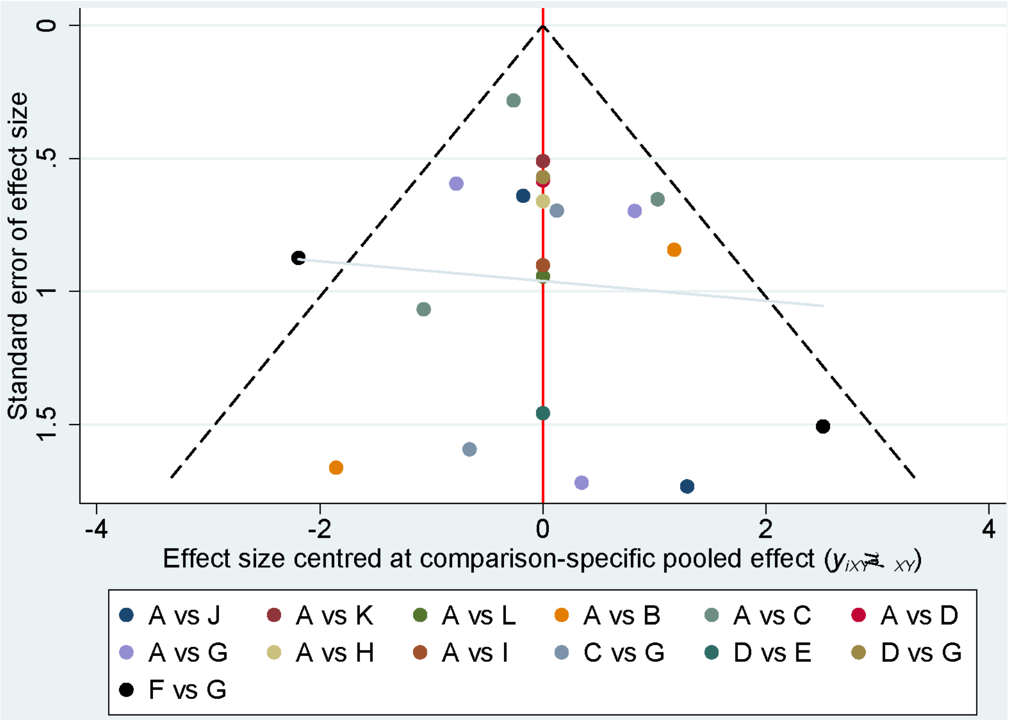

Supplementary FIGURE 43. The net-funnel of Primary Outcome-safety.
